# Biocompatible nanocomposite hydroxyapatite-based granules with increased specific surface area and bioresorbability for bone regenerative medicine applications

**DOI:** 10.1038/s41598-024-79822-0

**Published:** 2024-11-15

**Authors:** Marta Trzaskowska, Vladyslav Vivcharenko, Aleksandra Benko, Wojciech Franus, Tomasz Goryczka, Adrian Barylski, Krzysztof Palka, Agata Przekora

**Affiliations:** 1 Department of Tissue Engineering and Regenerative Medicine, Medical University of Lublin, Chodzki 1, 20-093 Lublin, Poland; 2 Faculty of Materials Science and Ceramics, AGH University of Science and Technology, Mickiewicza 30, 30-059 Krakow, Poland; 3 Department of Construction Materials Engineering and Geoengineering, Lublin University of Technology, Nadbystrzycka 38 D, 20-618 Lublin, Poland; 4 Institute of Materials Engineering, University of Silesia in Katowice, 75 Pułku Piechoty 1A, 41-500 Chorzów, Poland; 5 Department of Materials Engineering, Lublin University of Technology, Nadbystrzycka 36, 20- 618 Lublin, Poland

**Keywords:** Biomaterials, Bone regeneration, Biopolymers, Bioceramics, Biocompatibility, Microhardness test, Biomedical materials, Biomaterials, Implants

## Abstract

Hydroxyapatite (HA) granules are frequently used in orthopedics and maxillofacial surgeries to fill bone defects and stimulate the regeneration process. Optimal HA granules should have high biocompatibility, high microporosity and/or mesoporosity, and high specific surface area (SSA), which are essential for their bioabsorbability, high bioactivity (ability to form apatite layer on their surfaces) and good osseointegration with the host tissue. Commercially available HA granules that are sintered at high temperatures (≥ 900 °C) are biocompatible but show low porosity and SSA (2–5 m^2^/g), reduced bioactivity, poor solubility and thereby, low bioabsorbability. HA granules of high microporosity and SSA can be produced by applying low sintering temperatures (below 900 °C). Nevertheless, although HA sintered at low temperatures shows significantly higher SSA (10–60 m^2^/g) and improved bioabsorbability, it also exhibits high ion reactivity and cytotoxicity under in vitro conditions. The latter is due to the presence of reaction by-products. Thus, the aim of this study was to fabricate novel biomaterials in the form of granules, composed of hydroxyapatite nanopowder sintered at a high temperature (1100 °C) and a biopolymer matrix: chitosan/agarose or chitosan/β-1,3-glucan (curdlan). It was hypothesized that appropriately selected ingredients would ensure high biocompatibility and microstructural properties comparable to HA sintered at low temperatures. Synthesized granules were subjected to the evaluation of their biological, microstructural, physicochemical, and mechanical properties. The obtained results showed that the developed nanocomposite granules were characterized by a lack of cytotoxicity towards both mouse preosteoblasts and normal human fetal osteoblasts, and supported cell adhesion to their surface. Moreover, produced biomaterials had the ability to induce precipitation of apatite crystals after immersion in simulated body fluid, which, combined with high biocompatibility, should ensure good osseointegration after implantation. Additionally, nanocomposite granules possessed microstructural parameters similar to HA sintered at a low temperature (porosity approx. 50%, SSA approx. 30 m²/g), Young’s modulus (5–8 GPa) comparable to cancellous bone, and high fluid absorption capacity. Moreover, the nanocomposites were prone to biodegradation under the influence of enzymatic solution and in an acidic environment. Additionally, it was noted that the hydroxyapatite nanoparticles remaining after the physicochemical dissolution of the biomaterial were easily phagocytosed by mouse macrophages, mouse preosteoblasts, and normal human fetal osteoblasts (in vitro studies). The obtained materials show great potential as bone tissue implantation biomaterials with improved bioresorbability. The obtained materials show great potential as bone tissue implantation biomaterials with improved bioresorbability.

**Supplementary Information:**

The online version contains supplementary material available at 10.1038/s41598-024-79822-0.

## Background

Nowadays, there is a high clinical demand for novel biocompatible materials and scaffolds used for bone tissue regeneration. Novel biomaterials are most often composed of calcium phosphates, which imitate the inorganic bone part, and polymers, which mimic the organic flexible matrix of the bone^1^. In the specialized literature, hydroxyapatite (HA), tricalcium phosphates, and biphasic calcium phosphates are the most used and predominant calcium phosphates^2^. Human bone consists of up to 70% hydroxyapatite, 25% organic matter, and 5% water, and thus, HA has attracted the widest interest z. HA has excellent biocompatibility, high bioactivity, non-toxicity, and osteoconductivity^3^. HA-based biomaterials can be produced in different forms, such as granules, and solid or porous scaffolds to fill small bone defects and stimulate the regeneration process. Importantly, HA granules are still the most frequently used biomaterials in orthopedics, stomatology, and maxillofacial surgeries to fill small bone defects and stimulate the regeneration process. Appropriate HA synthesis factors, such as pressure or temperature, and their intensification determine the microstructure of HA ceramics, which in consequence also translates to their properties^4^. Depending on the selected sintering temperature, hydroxyapatite exhibits completely different properties. Low sintering temperatures (< 900 °C) procure high ionic reactivity and reduce the biocompatibility of HA, causing a cytotoxic effect under in vitro conditions, simultaneously resulting in high bioactivity, good bioresorption, high specific surface area (SSA), and high porosity of the HA^5–9^. The opposite scenario is observed when a higher (≥ 900 °C) sintering temperature is selected. As a result of applied high temperatures during the sintering process, improved biocompatibility of the resultant HA is observed. Still, this type of bioceramics is characterized by inferior microstructural properties (especially, low porosity and SSA) compared to the HA sintered at low temperatures^9–13^. Consequently, high sintering temperature results in “super stable” and highly crystalline HA with limited microstructural features^10,12,14^. All mentioned above negatively affect the bioactivity, osseointegration, and bioresorbability of the HA sintered at high temperatures An attempt to improve the properties of calcium phosphate ceramics was the production of biphasic calcium phosphates (BCP) by mixing stable hydroxyapatite and soluble β-tricalcium phosphate (β-TCP)^15^. The advantage of this type of ceramics is the ability to control bioactivity and the degree of biodegradation that affects the stability of the biomaterial and osseointegration. However, two-phase ceramics have poor mechanical properties, making them unsuitable for use in load-bearing implantation areas. Importantly, the optimal ratio of HA to β-TCP has not been reported so far^16^. Another strategy to obtain hydroxyapatite with improved bioresorbability and high specific surface area was the production of biomimetic ceramics. In this method, calcium phosphate is formed as a result of precipitation and dissolution reactions at low temperatures, imitating the phenomenon of biomineralization^17^. Nevertheless, in this case, the production process is lengthy, the solutions in which it takes place has to be frequently replaced, and its pH has to be maintained at a constant, very specific level^18^. Hence, this process is unfavorable from the economic point of view. Therefore, it seems reasonable to develop a method to produce a biomaterial based on hydroxyapatite that would provide the best properties supporting the bone repair process. An important difference between calcium-phosphate ceramics and natural bone tissue is the presence of an organic phase in the bone. Bone is a natural composite in which the polymer part improves the bioactivity and the elasticity of the tissue^19^. According to the latest scientific reports, the use of natural and synthetic polymers for the production of composite hydroxyapatite-polymer materials is of great interest^2,20–22^. Chitosan is often used as a component of bone biomaterials due to its high biocompatibility and ability to promote bone cell adhesion^23^. This chitin derivative also has antibacterial properties and possesses a beneficial effect on the biodegradation process^24,25^. Another ingredient characterized by high biocompatibility and lack of immunogenicity is agarose. By altering the concentrations of agarose, it is possible to adjust the mechanical properties and permeability of the biomaterial^26,27^. Curdlan (bacterial β-1,3-glucan), on the other hand, is a biocompatible polymer which due to its unusual gelling properties may provide good mechanical strength of the biomaterial. This specific β-1,3-glucan creates two types of gels with different properties, depending on the gelling temperature used^28,29^.

The present work aimed to develop nanocomposite HA granules (nano-filled polymer composites) that would reveal osteoconductive properties similar to HA sintered at high temperatures and possess appropriate microstructural features comparable to HA sintered at low temperatures. To produce nanocomposite material, nanohydroxyapatite (nanoHA) sintered at a high temperature (1100 °C) was used and combined with chitosan-based (curdlan/chitosan and agarose/chitosan) matrix that served as a binder for nanoHA. NanoHA-based granules were fabricated using a gas-foaming agent combined with a lyophilization technique to improve the porosity and specific surface area (SSA) of the resultant biomaterial. Chitosan, agarose, and curdlan (linear β-1,3-glucan) were selected due to their non-toxicity and high biocompatibility^26,30,31^. They are green-derived and abundant polysaccharides, so their usage fits well into the ecological objectives of the current world. The presence of chitosan in each granulate was supposed to improve its osteoconductive properties (stimulation of adhesion and growth of osteoblasts). Agarose and curdlan, on the other hand, were preferred mainly for their ability to create compact and solid gels. This feature is intended to improve the mechanical properties of the biomaterials. This study was designed with an aim to select the most favorable mixture of polysaccharides. In particular, curdlan has the unique property of forming a dense and resilient gel at high temperatures (> 80 °C) [36]. Also, hydroxyl groups of agarose or curdlan should be able to form chemical interactions with amino groups of chitosan molecules, leading to the formation of a hybrid matrix, characterized by improved stability and mechanical properties compared to pure chitosan. The ability of the two polysaccharides to interact with one another has already been proven in our recent studies^31,32^. In this research, 2 types of granulates were produced that had either chitosan/agarose or chitosan/curdlan matrix. By comprehensive evaluation of the microstructural and biological properties of these granulates, it was possible to gain knowledge of which biopolymer with gelling ability is better for the production of biomaterials with good biocompatibility and mechanical parameters.

## Methods

### Preparation of the granules

**Fig. 1:**
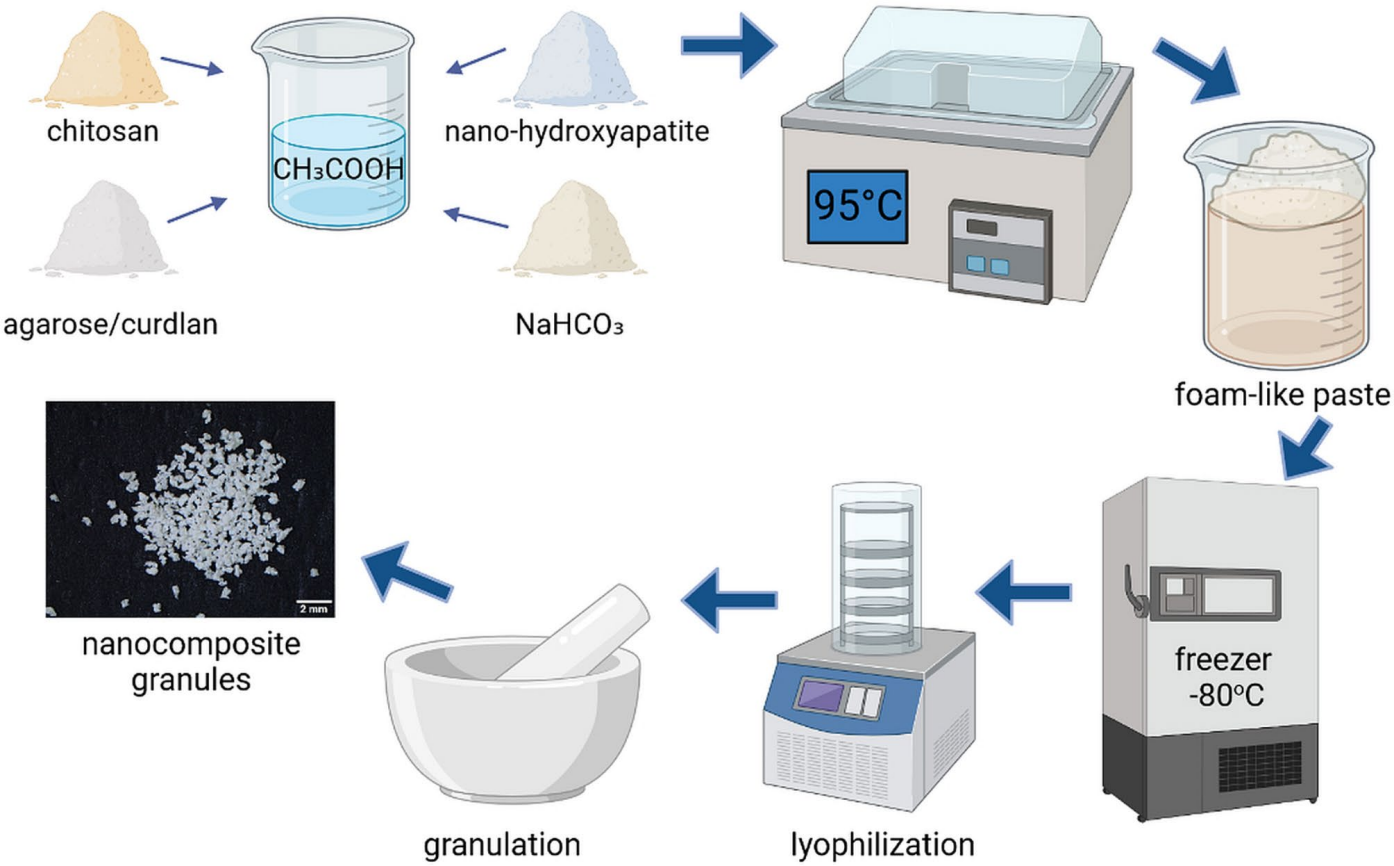
Production scheme of polymer-hydroxyapatite nanocomposite granules.

Nanocomposite granules were synthesized according to Polish patent application No. P.442,451 (2022). Firstly, a 2% v/v acetic acid (CH_3_COOH) solution in distilled water (Avantor Performance Materials) was prepared. Then, the following ingredients were added to 10 mL of CH_3_COOH solution: 200 mg chitosan (50–190 kDa molecular weight, 75–85% deacetylation degree, Sigma-Aldrich Chemicals), 500 mg agarose (low EEO, gel point 36 ± 1.5 °C, Sigma-Aldrich Chemicals) or 400 mg curdlan (DP 6790; specific rotation: [α]D20 = + 30°~ + 35°; gel stability: pH 2.0– 9.5 with max. gel strength: pH 2.0– 3.0, Wako pure Chemicals Industries), and 4 g nanohydroxyapatite powder (particle size < 200 nm, SSA ≥ 9.4 m^2^/g, Sigma-Aldrich Chemicals). Applied here amounts of chitosan, agarose, and curdlan were fixed to be 200 mg, 500 mg, and 400 mg per 10 mL of CH_3_COOH solution, respectively, based on the optimization process presented in our previous research. These concentrations of the polymers were proven to provide appropriate microstructural and biological properties^33–35^. Then, 200 mg of sodium bicarbonate (Sigma-Aldrich Chemicals) were added to each resultant paste made of biopolymers and hydroxyapatite, and mixed. This was then transferred to a mold and subjected to the incubation process at 95 °C for 20 min. After the incubation, the obtained materials were frozen at − 80 °C and freeze-dried (Martin Christ, lyophilizer model: Alpha 1–4 LSC basic) for a period of 19 h. Lyophilized samples were next soaked in PBS solution for 90 min and dried at room temperature for 24 h. The dry mass of chitosan/agarose/nanoHA and chitosan/curdlan/nanoHA samples contained individual components at the concentrations 4.3%/10.6%/85.1% and 4.4%/8.6%/87% by weight, respectively. To obtain granules with a diameter in the range of 0.3–0.4 mm, the samples of the biomaterials were granulated in a mortar and the fraction formed between sieves (# 0.300 and # 0.400, no 97179 and no 96847, respectively, Multiserw-Morek, IS0 3310-1) with a mesh diameter of 0.3 mm and 0.4 mm was collected. The working height of the sieve was 25 mm. The chitosan/agarose/nanoHA biomaterial is marked as chit/aga/nanoHA whereas chitosan/curdlan/nanoHA biomaterial is referred to as chit/curd/nanoHA. A simplified biomaterial production scheme is presented in Fig. 1.

The granules used in the tests were sterilized with ethylene oxide. Within the studies, two reference biomaterials were used: (1) commercially available hydroxyapatite granules sintered at high temperature of 1250 °C (HA BIOCER, Chema Elektromet Rzeszow, Poland; marked as HA high ST) and (2) hydroxyapatite granules sintered at low temperature of 400 °C, produced by wet chemical precipitation method (marked as HA low ST). The crystallinity phase composition of the produced HA low ST and the commercial hydroxyapatites (HA BIOCER and nanoHA) was confirmed using the X-ray diffraction analysis (XRD, HZg-4 Carl Zeiss Jena). The XRD proved the purity phase of all bioceramics used. HA low ST, as expected, had the smallest crystallite size of approximately 9.92 nm, whereas the crystallite size of nanoHA and HA high ST (HA BIOCER) was equal to 50.77 nm and 50.78 nm, respectively, which is typical of hydroxyapatite sintered at high temperatures^36^ (Supplementary Data 1).

### Physicochemical properties of the granules

The chemical composition of the samples was evaluated via Fourier transform infrared spectroscopy (FTIR) operating in a transmittance mode (Bruker Tensor 27). Around 2 mg of each sample was separately mixed with approximately 200 mg of potassium bromide (KBr, Merck, pre-dried before the experiments in vacuum drier (Vacucell 55) at 110°C for at least 12 h) and compressed into pellets using 13 mm evacuable pellet dies (Specac). 64 scans, recorded with 4 cm^− 1^ resolution in the medium infrared region (4000–400 cm^− 1^), were accumulated for each measurement and pure KBr pellet served as a background. Post-measurements, the baseline was manually corrected, and 9-point smoothing was performed in OPUS 7.2 software [https://www.bruker.com/en/products-and-solutions/infrared-and-raman/opus-spectroscopy-software.html].

To investigate the total amount of biopolymers surrounding the nanoHA granules and compare their thermal stabilities, a thermogravimetric investigation (TGA, NETZSCH, Jupiter F449) was conducted. When measuring in air, TGA gives a direct answer about the total amount of organic residue present in the sample (similar strategy is also employed for analyzing the amount of organic compound in the bone^37^). Second application relates to measurements conducted under the protective flow of nitrogen, wherein organic compound is not combusted, but instead – pyrolyzed and can reorganize. In this case, changes in the thermal stability are direct signs of chemical interactions between the compounds (and likely, the level of their crosslinking). Approximately 7 mg of each sample type was loaded, separately, into the concavus aluminum crucible, without a lid. Before measurements, correction was performed on an empty crucible. In two separate experiments, samples were heated from 40 to 500 °C, with a heating rate of 10 °C/min, under the protective flow of nitrogen (50 mL/min) or oxidizing synthetic air (50 mL/min). Using nitrogen flow allowed us to compare the thermal stabilities of the samples, while air was used to investigate the total amount of organic residue within the sample, assuming a complete combustion of the biopolymers up to 500 °C. Analysis of the obtained data was conducted in Proteus 6.1.0 software [https://analyzing-testing.netzsch.com/en/products/software].

Prior to TGA and FTIR analysis, the samples were placed in a vacuum drier (Vacucell 55) set to 40^o^C for at least 24 h to remove any residual moisture or physisorbed water. The measurements were conducted right after the samples were removed from the drier to reduce the chance of the hygroscopic effect.

Results of the FTIR and TGA analysis were visualized using Origin 2021. FTIR spectra were maximized and offset for better clarity.

### Microhardness testing

Micro Combi Tester (MCT3 microhardness tester, Anton Paar, Corcelles-Cormondrèche, Switzerland) was used in the evaluation of the granules plane strain modulus of elasticity. The conducted test was carried out according to the ISO 14,577 standard [ISO14577-1:2015]. Samples were embedded in resin and polished, whereupon all measurements were conducted using a Berkovich diamond indenter (SN: B-V83; α = 65.3° +/− 0.3°, maximum load 50 mN). The indenter loading and unloading speed was 100 mN/min. To determine Young’s modulus, indentation curves (the relationship between normal load and penetration depth) were measured during the microhardness test. In the experiment, three independent samples of each biomaterial were tested.

### Microstructure characterization

Specific surface area measurements of the obtained granulates were conducted with the use of the nitrogen adsorption technique with the Brunauer-Emmett-Teller theory. The granules’ porosity (area occupied by pores) and average pore diameter were evaluated by the mercury intrusion porosimetry method (AutoPore IV 9500 porosimeter, Micromeritics). The microstructures of the granules’ surface were visualized using a stereoscopic microscope (Olympus SZ61TR) and scanning electron microscope (SEM; JEOL JCM-6000Plus or FEI Nova NanoSem 450). Before SEM analysis, all samples were coated with a 15 nm gold layer. The SEM images were acquired at an accelerating voltage of 5 kV. Three independent samples of each biomaterial were tested in the experiments.

### Bioactivity analysis

Spontaneous apatite formation on the produced granules was determined in accordance with the ISO 23317:2012 procedure, as described earlier^38^. Briefly, after 14 days of the sample incubation in a simulated body fluid (SBF) at 37 °C, apatite crystals that formed on the granules’ surfaces were visualized using SEM (JEOL JCM-6000Plus or FEI Nova NanoSem 450), equipped with an Octane Pro EDS (energy dispersive spectroscopy) detector (EDAX, Carl Zeiss Microscopy). The Ca/P atomic ratio was calculated based on EDS data collected after the apatite precipitation. During the test, three independent samples of each biomaterial were examined.

### Biodegradation assay

The biodegradation of the produced granules was estimated in different enzymatic and non-enzymatic solutions. An enzymatic biodegradation test was carried out according to the method described by Gorgieva et al. and Kazimierczak et al. with some modifications^33,39^. To assess the susceptibility of the granules to the enzyme-mediated bone remodeling process, the samples were weighted (20 ± 2 mg) and immersed in the enzymatic lysozyme/type I collagenase solution (1 g/L and 150 mg/L, respectively). Lysozyme was purchased from Sigma-Aldrich Chemicals, whereas Type I collagenase was from Thermo Fisher Scientific. To determine the susceptibility of the granules to the osteoclast-mediated bone remodeling process related to the lowering of pH, the samples were placed in a non-enzymatic HCl solution of pH 4.5 prepared in distilled water. The stability of the granules was checked in a non-aggressive isotonic microenvironment i.e. 0.9% NaCl solution. The granules were incubated in degradation solutions for 8 weeks at 37 °C, with agitation (60 rpm). The degree of degradation process was estimated based on the sample weight loss after the incubation period. Three independent samples of each biomaterial were tested in the study.

### Bioresorbability evaluation

The protocol for this test was developed by our research team and formed based on the following knowledge: (1) Alizarin Red S can bind with calcium phosphates and can be visualized in a confocal microscope^40,41^; (2) The sediment formed after the degradation of the biomaterial contains, among others, hydroxyapatite nanoparticles; (3) Osteoclasts and macrophages can phagocytize hydroxyapatite nanoparticles^42,43^. For a bioresorbability test, HA nanopowder used for biomaterial production was stained with 2% (w/v) Alizarin Red S (ARS) (Sigma-Aldrich Chemicals). Then, the granules were produced using stained nanoHA. Afterward, 200 mg of synthesized samples were incubated in phosphate-buffered saline (PBS, Sigma-Aldrich Chemicals) without calcium and magnesium for 8 weeks. After incubation, PBS was collected, and centrifuged and the resulting pellet containing stained HA nanoparticles (released from the granules due to sample degradation) was suspended in a medium dedicated to the used cell line (see details in the section Cell culture experiments). The bioresorption of the degradation product of the granules was estimated using 3 different cell lines: normal mouse calvarial preosteoblasts (MC3T3-E1 Subclone 4), human fetal osteoblasts (hFOB 1.19) and murine macrophage cell line, transformed with Abelson murine leukemia virus (RAW 264.7), purchased from ATCC (American Type Culture Collection, see section Cell culture conditions for culture maintenance details). Each cell line was seeded at a concentration of 1 × 10^5^ cells per well in a 48-multiwell plate and cultured for 24 h. Next, the cell culture medium was replaced with an earlier prepared nanoHA-containing medium, and cells were cultured for 2 days. Finally, the bioresorbability of produced biomaterial was evaluated by observation of each cell line under a confocal laser scanning microscope (CLSM) using additional Nomarski contrast (Olympus Fluoview equipped with FV1000).

### Absorption capacity evaluation

Human blood plasma derived from peripheral blood (informed consent was obtained from the volunteer; the study adhered to the tenets of the Declaration of Helsinki, the Bioethics Committee approval no. KE-0254/187/10/2022) and PBS with calcium and magnesium (PBS+) were used in the evaluation of granules’ absorption capacity. The samples with an average weight of about 15 ± 1 mg were immersed in 500 µL of the selected fluids for 1 h at room temperature. The fluid was poured from above the granules, the samples were wiped with filter paper to remove excessive plasma or PBS, and then the biomaterials were weighed using analytical balance. The granulates weight was checked at defined time intervals (2s, 8s, 16s, 30s, 60s, 120s, 300s, 600s, 900s, 1800s, and 3600s) and then the samples were immersed back into fluids. The maximum absorption capacity of the tested granules was calculated according to the equation described earlier^44^ The absorption capacity was presented as a percentage of weight increase (W_i_) over time, calculated based on the formula: W_i_=(W_t_ – W_0_)/W_0_ × 100, where W_t_ is biomaterial weight at time t, and W_0_ is the weight of the dry granules. In the experiment, three independent samples of each biomaterial were tested.

### Cell culture conditions

Cell culture experiments were conducted using mice and human osteoblasts. Mouse primary calvarial preosteoblast cell line (MC3T3-E1 Subclone 4, CRL-2593, ATCC-LGC standards) was cultured in Alpha Minimum Essential Medium (GIBCO, Life Technologies), supplemented with 10% fetal bovine serum (FBS, Pan-Biotech GmbH), 100 U/mL of penicillin and 0.1 mg/mL of streptomycin (Sigma-Aldrich Chemicals) at 37 °C, 95% of air humidity and 5% CO_2_. Normal human fetal osteoblast cell line (hFOB 1.19, ATCC-LGC standards) was cultured in DMEM/Ham’s F12 medium without phenol red (Sigma-Aldrich Chemicals), supplemented with 10% FBS, 300 µg/ml G418 (Sigma-Aldrich Chemicals), 100 U/mL of penicillin and 0.1 mg/mL of streptomycin, and incubated at 34 °C, 95% of air humidity and 5% CO_2_. Murine macrophage cell line transformed with Abelson murine leukemia virus (RAW 264.7, ATCC-LGC standards) was cultured in Dulbecco’s Modified Eagle’s Medium (DMEM, ATCC-LGC standards) supplemented with 10% FBS, 100 U/mL of penicillin and 0.1 mg/mL of streptomycin at 37 °C, 95% of air humidity and 5% CO_2_.

### Cytotoxicity and cell growth evaluation

The indirect cytotoxicity test was carried out in accordance with the procedure described in ISO 10993-5 ^45^ using biomaterial extracts, prepared according to ISO 10993-12 ^46^. The extracts were prepared by submersing 100 mg of each biomaterial in 1 mL of cell culture medium for 24 h at 37 °C. To assess the cytotoxicity, MC3T3-E1, and hFOB 1.19 cells were seeded in 100 µL of the medium at a concentration of 2 × 10^5^ cells per mL in 96-multiwell plates and cultured for 24 h. After that, the cell culture medium was replaced with granule extracts, and cells were cultured for the next 24 h. After 24 h of incubation, an MTT test was performed to evaluate cell viability. Cytotoxicity assay was conducted in accordance with a procedure described previously^13^. The test was conducted in three independent repetitions and the obtained results were presented as percentages of the absorbance value of the negative control (cells maintained in polystyrene extract). The cytotoxicity of the produced granules was also estimated by a direct-contact method. Briefly, 1 × 10^5^ of MC3T3-E1 and hFOB 1.19 cells were cultured directly on the granules placed in a 24-well plate for 72 h, stained using Live/Dead double fluorescent staining kit (Sigma-Aldrich Chemicals) and observed under CLSM (Olympus Fluoview equipped with FV1000). Viable cells were stained with calcein-AM and possessed strong green fluorescence, while dead cells were stained with propidium iodide (PI) and possessed red fluorescence.

### Statistical analysis

All obtained results were presented as mean values ± standard deviation (SD). For the cytotoxicity test, statistically significant differences between control cells maintained in the polystyrene extract and cells grown in the granules extracts were considered at *p* < 0.05 according to a One-way ANOVA with post-hoc Dunnett’s test (one fixed control group was compared to all of the other samples). For other tests, a One-way ANOVA, followed by Tukey’s test, with statistically significant results considered at *p* < 0.05, was applied to analyze statistical differences between all groups (all materials were compared with each other). Statistical analysis of the obtained data was performed using GraphPad Prism 8.0.0 Software [https://www.graphpad.com/] (GraphPad Software Inc.).

## Results

### Physicochemical properties of the granules

**Fig. 2:**
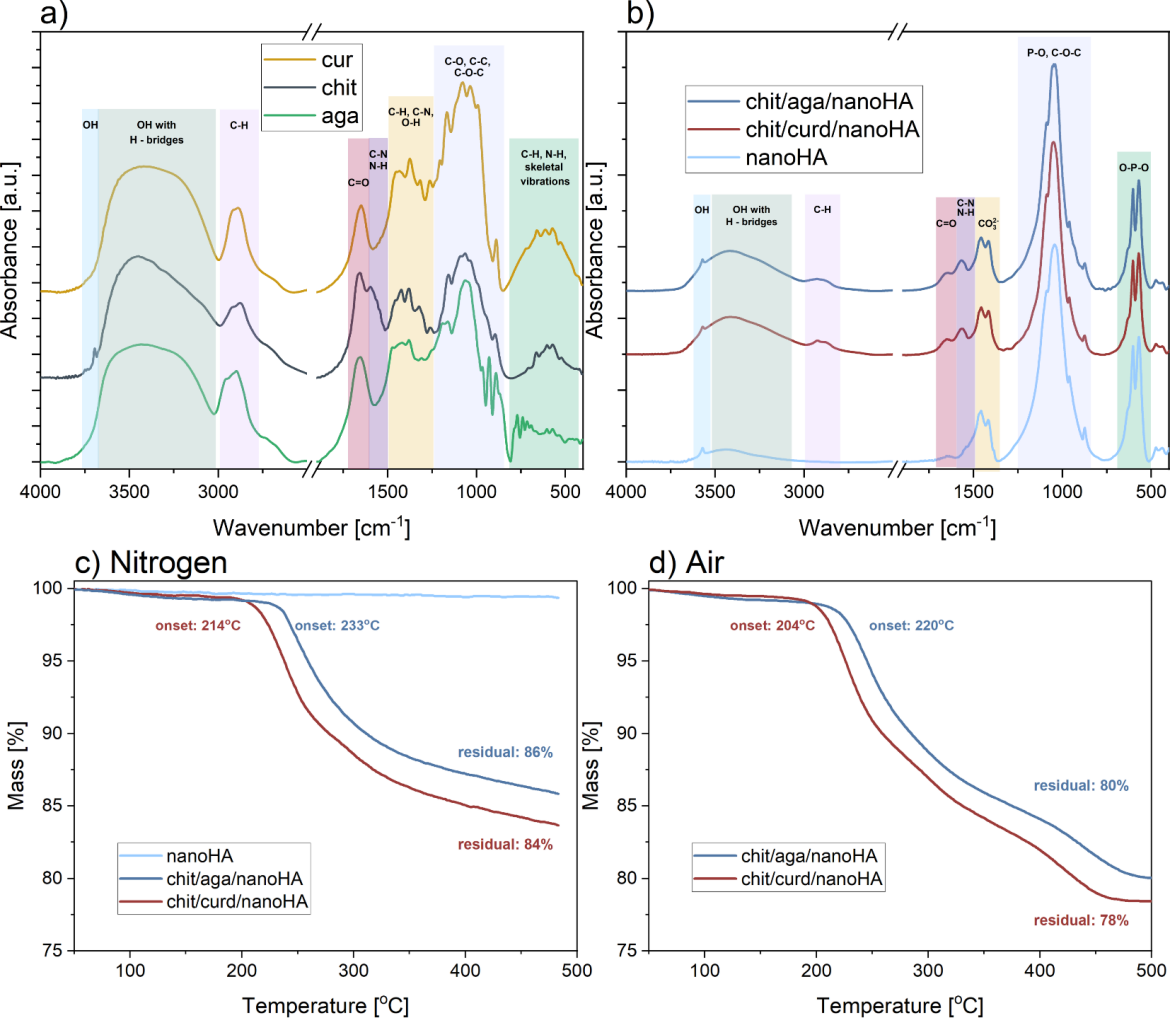
Physicochemical properties of the granules: (**a**) FTIR spectra of the chitosan, agarose, and curdlan used in this study, (**b**) FTIR spectra of the nanoHA and its nanocomposites, (**c**) and (**d**) thermogravimetric analysis (TGA) conducted in nitrogen and synthetic air, respectively. FTIR spectra were offset and maximized for better clarity, and the characteristic vibrational modes were marked with colors.

Curdlan, chitosan, and agarose are all polysaccharides – their chemical structures are very similar to one another, with the main constituent being a repeating 5-carbon ring. In the case of chitosan and curdlan, this ring is glucose, while in agarose: glucose’s isomer, galactose. Curdlan has the simplest chemical formula of the three, with repeating β-(1 → 3)-linked glucose units. In chitosan, the glucose ring has side chain functional groups: n-acetyl or amine. Meanwhile, in agarose, D-galactose rings are connected to 3,6-anhydro-L-galactose units. Details of the chemical structures and vibrational modes present in the compounds can be found in our previous studies^32,47^. Having similar chemical structures, these polysaccharides also had similar FTIR spectra. Still, some hallmarks of each material could be identified (Fig. 2a). To better highlight these, additional, maximized and superimposed spectra were plotted and these are presented in the Supplementary Data 2. In chitosan, some of the most characteristic were the features attributed to amines/amides functional groups – the presence of the amide II attributed bonds (C-N and N-H) at 1597 cm^− 1^ and sharpening of the OH-attributed bonds (between 3000 and 3700 cm^− 1^), with a small shoulder at 3695 cm^− 1^, indicative of OH groups not bonded through hydrogen bridges. Because of the presence of 3,6-anhydro-L-galactopyranose unit in agarose, its spectrum was characterized by broadening of the triplet between 2800 and 3000 cm^− 1^ (attributed to C-H bonds), and significantly more vibrational modes observed below 1000 cm^− 1^ (single bonds between carbon, nitrogen, and hydrogen).

Investigating the FTIR spectra of the nanocomposites, one can tell that the signal was mostly dominated by the bands attributed to the hydroxyapatite (Fig. 2b). These are: a high-intensity composite band with a peak at 1044 cm^− 1^, which was attributed to the stretching of the P-O bonds^48^; medium-intensity doublet with peaks at 603 and 570 cm^− 1^, originating from bending of the O-P-O bonds in the crystalline apatite^48^; two small-intensity bands at 1457 and 1417 cm^− 1^ from CO_3_^2−^ groups, characteristic of biological HA (but also a possible reaction by-product from sodium bicarbonate used in the nanocomposites preparation)^49^, and two peaks attributed to OH groups: one wider due to hydrogen bridges, and one sharp due to unbound OH (characteristic of HA)^50^. Still, some minute but distinctive changes to the spectra could be identified with an addition of both types of blends: chitosan + agarose or chitosan + curdlan. To better highlight these, additional, maximized and superimposed spectra were plotted and these are presented in the Supplementary Data 2. There was an increased intensity of the OH-attributed bonds and the appearance of new bonds: between 3000 and 2800 cm^− 1^, coming from the C-H bonds in alkanes (inside the saccharide ring), and between 1700 and 1500 cm^− 1^, due to C = O and C-N/N-H (amide II in chitosan) stretching, respectively. Herein, altered relative intensities of the two latter bands indicated a mixture of chitosan and another polysaccharide. In chit/aga/nanoHA, a triplet identified in the 3000 and 2800 cm^− 1^ region was wider, which was characteristic of agarose (Fig. 2a). Meanwhile, in chit/curd/nanoHA, two sharper peaks were better defined, indicative of both curdlan and chitosan. In chit/aga/nanoHA, there was also a set of small-intensity bands observable between 950 and 650 cm^− 1^, which originated from substitution in alkenes/aromatic rings due to the presence of 3,6-anhydro-L-galactose in agarose. Additionally, when compared to the spectra of pure biopolymers, it was apparent that the shape of the OH-attributed band was altered, with maxima shifted towards lower wavenumbers, indicative of some chemical interactions involving hydrogen and oxygen atoms (most likely, hydrogen bridges between the biopolymers and between the biopolymer and HA, as suggested in our previous studies^31,38^). Due to the small contribution of the biopolymer-related FTIR bands, more specific chemical interactions that could classify materials as hybrids could not be identified. Yet, the presence of all types of polysaccharides used to fabricate the materials could be confirmed, suggesting that the adhesion of biopolymers to the inorganic granules was sufficiently strong to withstand autoclaving, grinding, and sieving.

To identify the thermal stability of the biopolymers, as well as their total amount, TGA analysis was conducted in nitrogen and air atmosphere (Fig. 2c and d, respectively). Under the protective nitrogen, mass loss of organic residues did not reach the plateau up to 500°C. Chit/curd/nanoHA was less stable than the chit/aga/nanoHA, initializing pyrolysis at a lower temperature (214 compared to 233°C), with mass loss peaking at 2.4%/°C compared with 1.9%/°C, and showing greater total mass loss of 16% as compared to 14%. Comparing the preparation step, one should highlight that in chit/aga/nanoHA a relatively larger total amount of biopolymers was used. Yet, in this sample, higher thermal stability of the used blend was reported. This might suggest that agarose was more thermally stable than curdlan, or that it formed a more crosslinked structure with chitosan than curdlan did. Both these suggestions could be true. An increased thermal stability is a typical hallmark of a more stabilized structure – usually, this means chemical interactions between the compounds, such as crosslinking^51,52^. However, agarose is also known as a carbonization precursor^53^, meaning that under certain conditions (typically, ambient atmosphere and two-step heating), carbon from its backbone can form a stable all-carbon structure. Because the experiments took place in air, carbonization is less likely (but not impossible). To further investigate this, TGA analysis in air was conducted. Heating the samples to 500^o^C in an oxidizing atmosphere of air should lead to a complete combustion of non-stabilized organic matter. This time, mass loss reached its plateau, with a residual mass of 80% reported in chit/aga/nanoHA and 78% found in chit/curd/nanoHA. Again, the onset of the thermal decomposition was higher for the chit/aga/nanoHA (204 compared to 220°C). All in all, these results suggest higher thermal stability of an agarose-based sample, possibly with improved crosslinking. Comparing residual masses, one could argue that there might have been more biopolymers in the chit/curd/nanoHA to start with. Still, this would not explain different onset temperatures, which are indicative of changes in the samples’ thermal stabilities, a hallmark of crosslinking.

### Mechanical and microstructural properties

In this study, a microhardness test was employed to determine stiffness of the biomaterials and it showed that HA low ST (reference HA sintered at a low temperature of 400 °C) and the chit/curd/nanoHA were characterized by slightly lower Young’s modulus (but without statistical significance), as compared to chit/aga/nanoHA. Unsurprisingly, HA high ST (reference HA sintered at a high temperature of 1250 °C), which had the lowest porosity among all samples, showed the highest Young’s modulus value (Table 1).

**Table 1:** Microstructural and mechanical parameters of the granules: porosity, average pore diameter, specific surface area (SSA), and plane strain Young’s modulus (E*).

Materials	Porosity [%]	Average Pore Diameter (µm)	SSA [m²/g]	E* [GPa]
HA high ST	26.7 ± 1.8 ^b, c,d^	28.4	2.4 ± 1 ^b, c,d^	82.0 ± 12.6
HA low ST	51.0 ± 1.5 ^a^	0.9	58.3 ± 0.3 ^a, c,d^	5.8 ± 0.9 ^a^
chit/aga/nanoHA	46.8 ± 2.1 ^a^	11.1	27.6 ± 0.2 ^a, b,d^	8.2 ± 1.9 ^a^
chit/curd/nanoHA	48.7 ± 0.9 ^a^	4.5	30.7 ± 0.4 ^a, b,c^	5.2 ± 1.7 ^a^

Statistically significant results compared with ^a^ HA high ST, ^b^ HA low ST, ^c^ chit/aga/nanoHA, ^d^ chit/curd/nanoHA, where p-value < 0.05; one-way ANOVA followed by Tukey’s test.

**Fig. 3:**
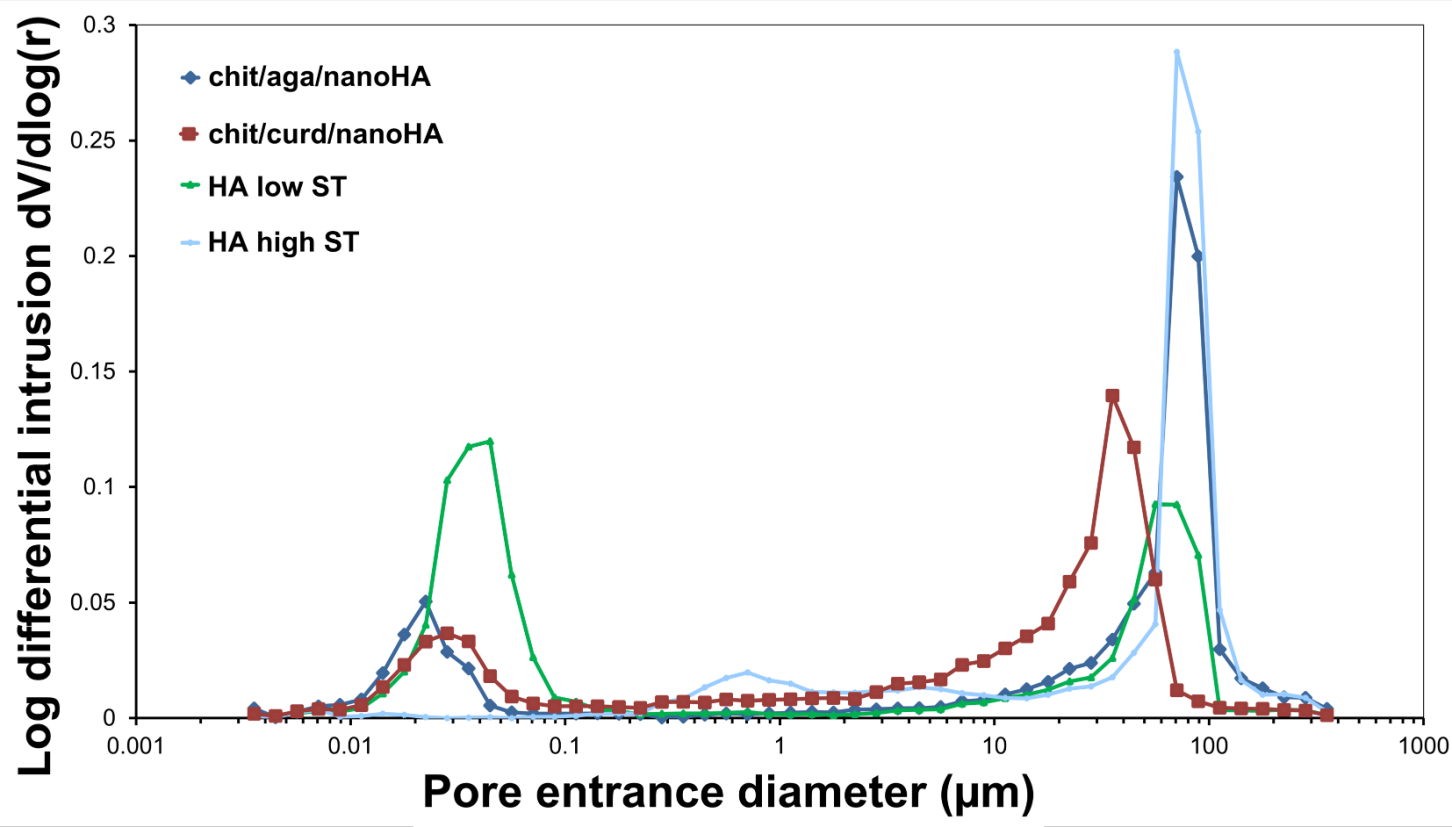
Pore size distribution of the produced granules (chit/aga/nanoHA, chit/curd/nanoHA) and reference samples (HA high ST and HA low ST).

Porosities of the developed granules were characterized by almost 2-fold higher values than HA high ST granules and were comparable to HA low ST (Table 1). The degree of porosity of the nanocomposite granules and HA low ST was also within the range of porosity characteristic of human cancellous bone (30–90%)^54^. Analysis of pore size distribution revealed that the microstructure of chit/aga/nanoHA was dominated by macropores, however, in contrast to HA high ST, the biomaterial also possessed a large amount of micropores, as can be seen in Fig. 3. In turn, chit/curd/nanoHA granules showed a large share of micro- and mesopores with the dominant presence of the latter. HA high ST possessed a negligible amount of micropores, whereas HA low ST was characterized by the highest content of micropores among all biomaterials, which was also reflected in their SSA values. Namely, HA high ST showed very low SSA while HA low ST exhibited the highest SSA among all samples (Table 1). This observation is in agreement with the available literature reports, which highlighted that a higher degree of porosity with the presence of small pores (meso- and primarily micropores) contributes to higher SSA of the biomaterial^55^. Produced nanocomposites had a relatively high SSA – higher compared to HA high ST, but approx. 2-fold lower compared to HA low ST. The obtained result can be explained by the lower share of micropores in the microstructures of produced biomaterials, as compared to the microstructure of HA low ST.

**Fig. 4:**
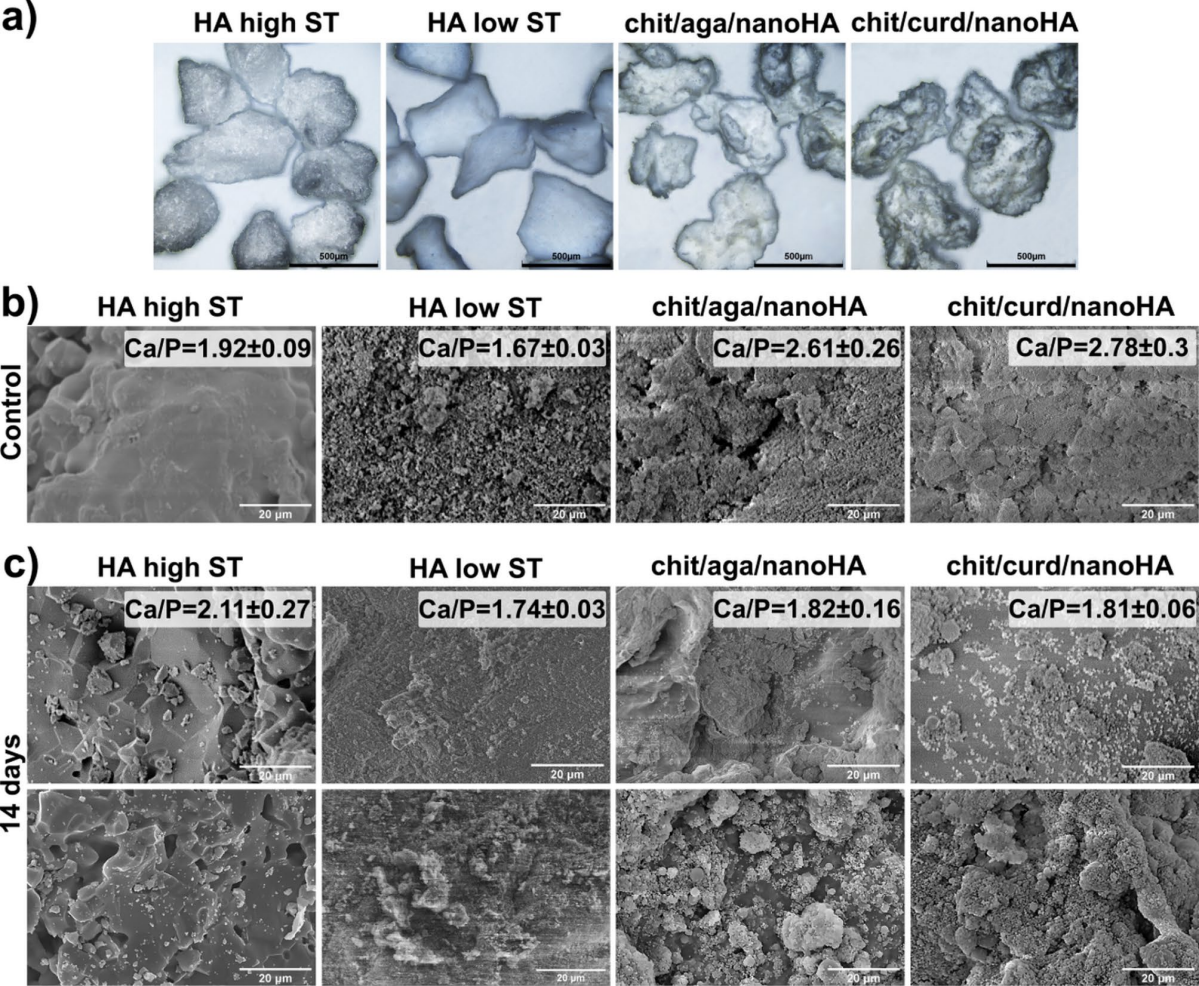
Visualization of the microstructure of the HA granules: (**a**) images obtained with a stereoscopic microscope, (**b**) granule surfaces imaged by SEM, (**c**) granule surfaces imaged by SEM after 14 days of soaking in SBF along with calculated Ca/P atomic ratio for precipitated apatite crystals.

Based on the images from a stereoscopic microscope, as-produced nanocomposite biomaterials were characterized by irregular and rough surfaces (Fig. 4a).To better visualize surface topography, a microstructure of the tested biomaterials was analyzed using SEM. Although the surface of HA low ST granules seemed to be relatively smooth on stereoscopic images (Fig. 4a), completely different results were obtained during SEM analysis. As can be seen in Fig. 4b, the chit/aga/nanoHA and chit/curd/nanoHA granules had a very rough surface, with numerous cracks and irregular edges, similar to HA low ST. Among the two nanocomposites, the surface of chit/curd/nanoHA appeared to be more like the surface of HA low ST (with smaller sizes of the features observable). Meanwhile, the surface of chit/aga/nanoHA had a higher share of larger features. These results are in line with the porosity evaluation (Table 1). In contrast, the use of a high sintering temperature in the production of HA high ST granules resulted in the formation of a dense biomaterial, with a compact and smooth surface.

### Bioactivity test

SEM imaging revealed that both chit/aga/nanoHA and chit/curd/nanoHA granules could induce the formation of apatite-like crystals on their surface. The resulting layer of crystals formed after 14-day incubation of the biomaterials in SBF had a spherical, hemispherical, or cauliflower-like shape that can be observed in Fig. 4c.

Based on the SEM-EDS analysis, it was demonstrated that the Ca/P atomic ratio of the crystals on the surface of chit/aga/nanoHA and chit/curd/nanoHA nanocomposites was equal to 1.82 and 1.81, respectively. Both the Ca/P values obtained for nanocomposites and HA low ST (1.74) granules were close to the value characteristic for natural stoichiometric hydroxyapatite (1.67)^33^. On the other hand, apatite crystals on the HA high ST had a Ca/P atomic ratio of 2.11, indicating the formation of a calcium-rich apatite layer. Calcium-rich apatite is an intermediate phase of apatite formation that is created by the interaction of negatively charged hydroxyl and phosphate groups on the surface of HA and the positively charged calcium ions from the solution. Subsequently, the interaction of the calcium-rich layer with the negatively charged phosphate ions from the solution results in the formation of a calcium-poor layer^56^. A higher Ca/P atomic ratio of HA high ST may indicate a slower course of the mineralization process, which is consistent with the research done by other authors^57,58^. Importantly, SEM-EDS analysis carried out for the samples before their immersion in SBF gave another evidence for the formation of the apatite layer on all biomaterials. Before incubation in SBF, spectra obtained for nanocomposite samples showed additional peaks for carbon elements derived from biopolymers. Immersion in SBF resulted in the disappearance of this peak in the EDS spectrum (Supplementary Data 3). Moreover, the Ca/P ratio for nanocomposite samples significantly decreased from approx. 2.7 to 1.8, proving changes in the number of atoms, with the relative amount of phosphate atoms increasing. This might suggest the mineralization process. In the case of reference samples, an opposite phenomenon occurred, immersion in SBF slightly increased the Ca/P ratio, indicating the formation of calcium-rich apatite.

### Assessment of biodegradation and bioresorbability

**Fig. 5:**
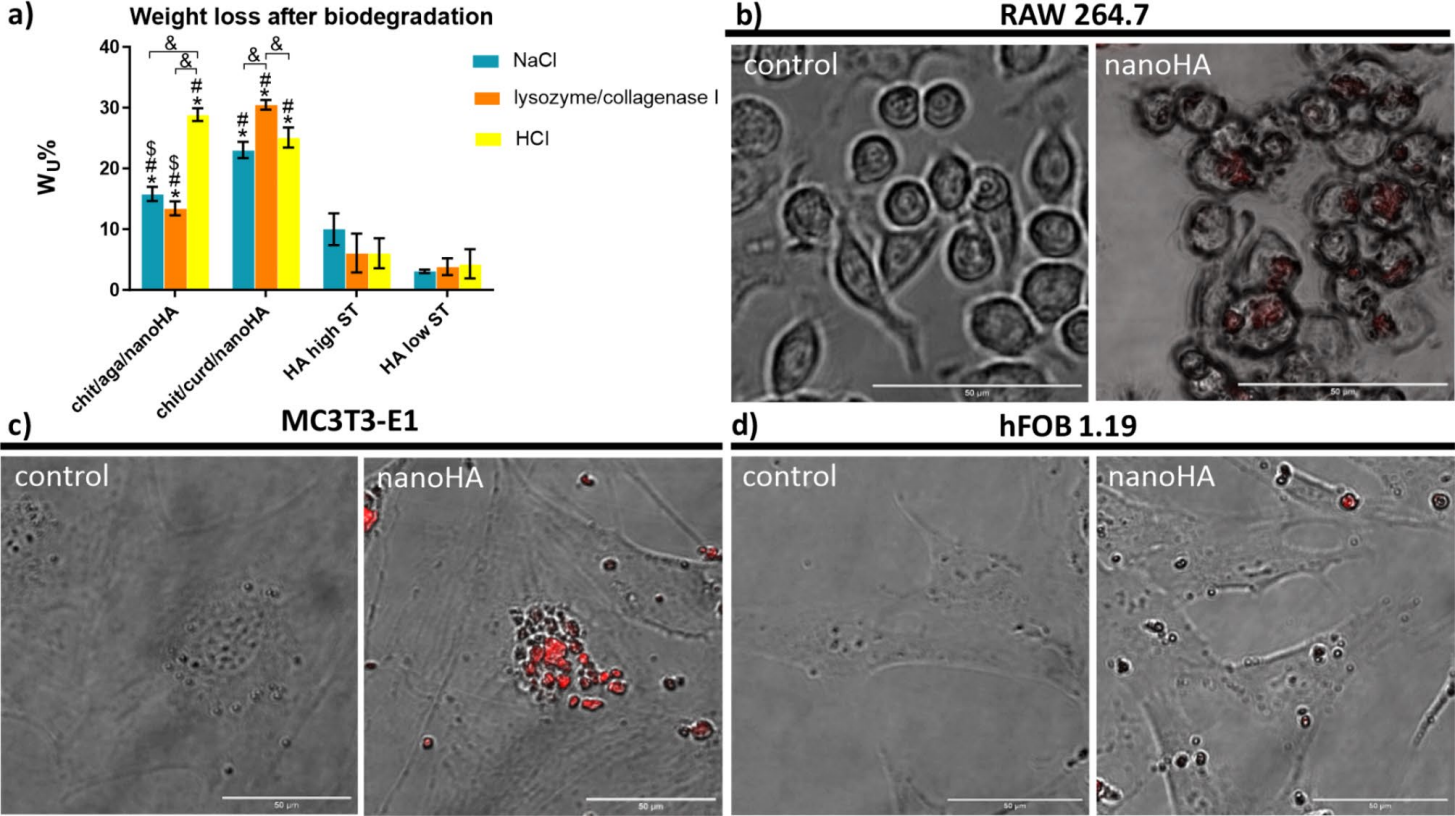
Assessment of biodegradation and bioresorption processes: (**a**) decrease in weight of the biomaterials (W_u_ %) after 8-week incubation in non-enzymatic NaCl and HCl solutions, and enzymatic lysozyme and collagenase I solution (* statistically significant results compared with HA high ST, ^#^ statistically significant results compared with HA low ST, ^$^ statistically significant results compared with chit/curd/nanoHA, ^&^ statistically significant results between indicated groups,* p*-value < 0.05; one-way ANOVA followed by Tukey’s test ); b-d) CLSM images showing uptake of released from biomaterial nanoHA (stained with Alizarin Red S) by different cell types: (**b**) mouse macrophages (RAW 264.7 cell line), (**c**) mouse preosteoblasts (MC3T3-E1 Subclone 4 cell line), and (**d**) human osteoblasts (hFOB 1.19 cell line); controls indicate control cells cultured in the absence of nanoHA.

Based on the results obtained, it was concluded that both nanocomposite granules degraded significantly faster compared to HA high ST and HA low ST (Fig. 5a). In the case of chit/aga/nanoHA, degradation in NaCl and enzymes led to around 15% mass drop which was consistent with the values obtained from the TGA analysis. This indicated that those conditions likely degraded only the biopolymer blend, not affecting the HA core. This was expected, as HA sintered at high temperatures should not degrade under the influence of enzymes that hydrolyze the peptide bonds (collagenase I) or (1→4)-β-linkages (lysozyme). HA should also be resistant to HCl and NaCl solutions. These results find reflection in the data reported for both types of reference HA, where low degradation values were reported, being also consistent with the literature^59,60^. Interestingly, after incubating in NaCl and enzymes, higher degradation rates were recorded for the chit/curd/nanoHA (20% and 30%, respectively) than the values found in TGA analysis (16%), indicating that not only did the polymers degrade, but also the HA core was affected. However, incubation of produced biomaterials (chit/aga/nanoHA and chit/curd/nanoHA) in HCl resulted in similar weight losses (approx. 25%).

In the images from the CLSM, HA nanoparticles were observed inside macrophages, mouse preosteoblasts, and human fetal osteoblasts (Fig. 5b–d), which confirmed the participation of these cells in the bioresorption of granules. In addition, cells incubated with HA nanopowder maintained their proliferation capacity and possessed normal morphology, which proved the lack of toxic effects of degradation products on eukaryotic cells.

### Assessment of fluid absorption capacity

**Fig. 6:**
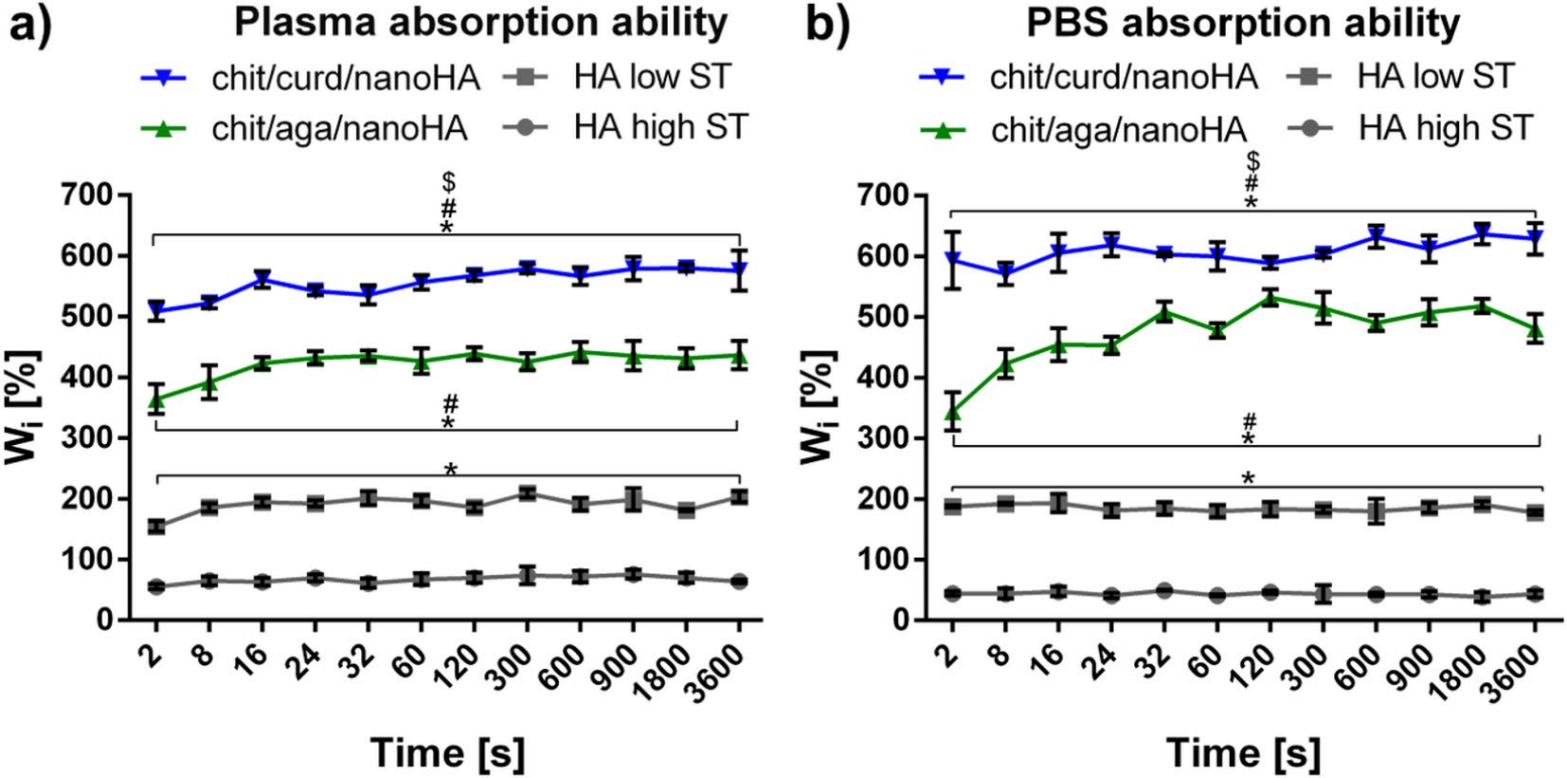
Evaluation of the liquid absorption capacity of biomaterials: (**a**) samples soaked in human blood plasma and (**b**) samples soaked in PBS; (* statistically significant results compared with HA high ST, ^#^ statistically significant results compared with HA low ST, ^$^ statistically significant results compared with chit/aga/nanoHA,* p*-value < 0.05; one-way ANOVA followed by Tukey’s test).

Two physiological solutions of different densities were used to test the liquid absorption capacity of granulates: human blood plasma and PBS. The test results showed that both chit/aga/nanoHA and chit/curd/nanoHA biomaterials could absorb a large volume of liquid in a very short period of time (Fig. 6a and b). Due to the higher density of blood plasma compared to the PBS, a slightly greater absorption capacity of biomaterials was observed in the latter solution. The chit/aga/nanoHA and chit/curd/nanoHA granules reached equilibrium after 32 and 24 s of soaking in PBS, respectively (W_i_ = 508.9% ± 16.4%, W_i_= 618.9% ± 19%) (Fig. 6b). In the case of blood plasma, agarose-based and curdlan-based biomaterials reached equilibrium after 32 and 16 s of soaking respectively (W_i_ = 435.6% ± 8.4%, 561.1% ± 13.9%, respectively) (Fig. 6a). Both reference samples (HA high ST and HA low ST) absorbed significantly fewer liquids than developed biomaterials. Unsurprisingly, HA low ST, characterized by high porosity and SSA, revealed a higher absorption capacity than the HA high ST of low porosity and SSA.

### Cytotoxicity and cell growth

**Fig. 7:**
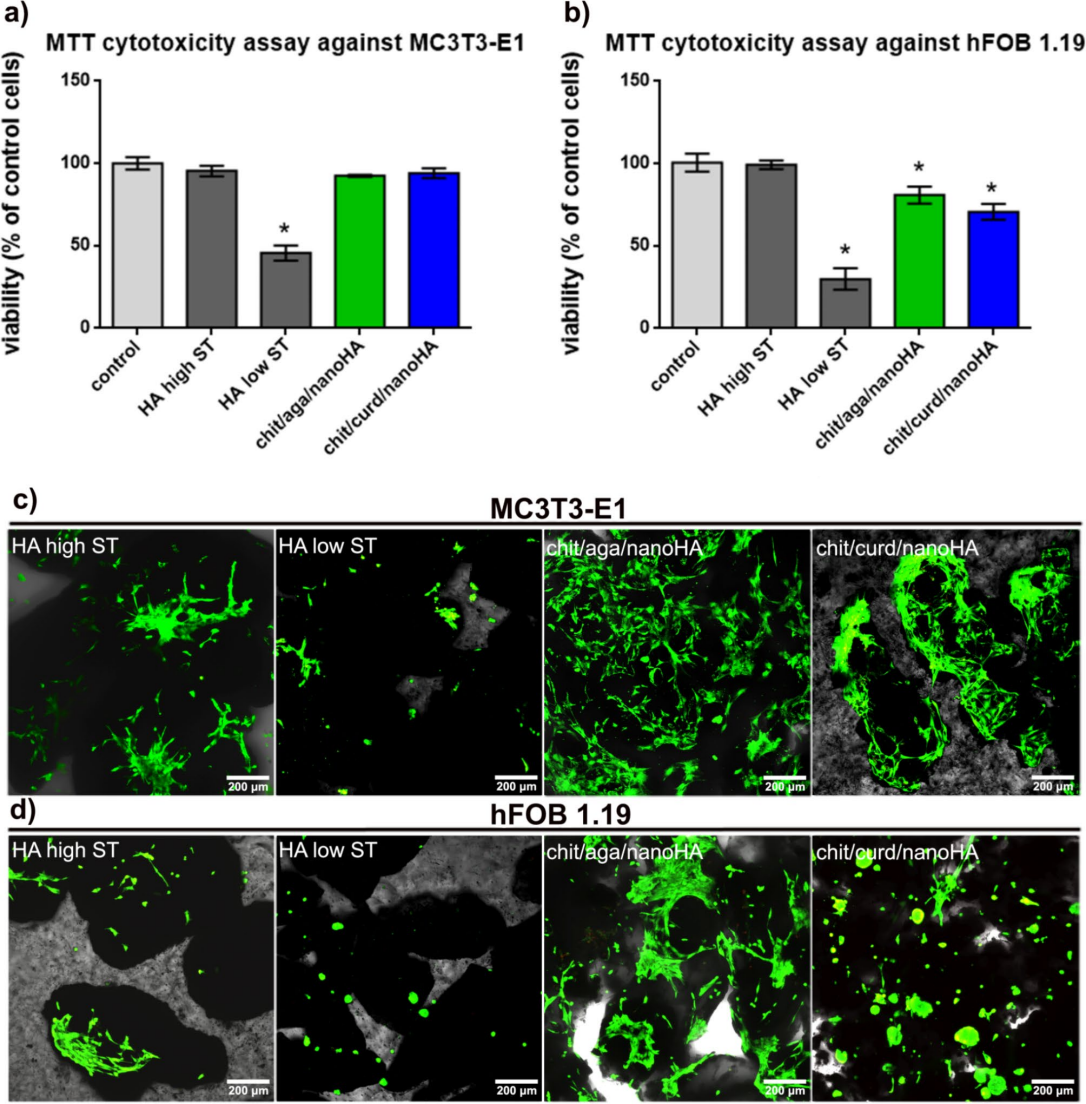
Biocompatibility tests on developed granules: (**a**) and (**b**) indirect MTT cytotoxicity assay against mouse preosteoblasts (MC3T3-E1 cell line) and human fetal osteoblasts (hFOB 1.19 cell line), performed according to ISO 10993-5 standard (* statistically significant results compared with control cells with* p*-value < 0.05; one-way ANOVA followed by Dunnett’s test); (**c**) and (**d**) cell adhesion, viability, and growth visualized by confocal laser scanning microscope after Live/Dead staining (viable cells emitted green fluorescence and dead cells red fluorescence, magnification 100×; scale bar = 200 μm).

In vitro studies showed that the nanocomposite granules and HA high ST were non-toxic to mouse preosteoblasts (MC3T3-E1) and human fetal osteoblasts (hFOB 1.19). Cell viability in the MTT assay after 24-hour exposure to the chit/aga/nanoHA and chit/curd/nanoHA granulate extracts was 92.4% ± 0.6% and 94% ± 3.1% for MC3T3-E1 cells and 80.8% ± 5.1% and 70.6% ± 4.8% for hFOB 1.19 cells, respectively (Fig. 7a and b).

Both types of nanocomposite granulates (despite a statistically significant decrease in the viability of hFOB 1.19 cells compared to the control) were classified as non-cytotoxic biomaterials according to the ISO standard since the cell viability did not decrease below 70%. Unlike nanocomposite granules and HA high ST, HA low ST significantly reduced the viability of both MC3T3-E1 and hFOB 1.19 cells to 45.6% ± 4.6% and 30% ± 6.5%, respectively. Thus, this sample exhibited a cytotoxic effect in the MTT assay. The lack of cytotoxicity of the nanocomposite biomaterials was confirmed by Live/Dead staining. Well-attached, flattened, and viable (emitting green fluorescence) cells of the MC3T3-E1 and hFOB 1.19 lines were observed on the surface of chit/aga/nanoHA granules (Fig. 7c and d). Viable and flattened mouse preosteoblast cells were also noted on the chit/curd/nanoHA biomaterial (Fig. 7c). However, noticeably fewer human osteoblasts (hFOB 1.19 cells) adhered to the chit/curd/nanoHA granules compared to the agarose-based sample (Fig. 7d). Importantly, the cell density observed on the chit/aga/nanoHA was higher than on the surface of HA high ST - material which is regarded as highly biocompatible and supportive for cell growth. On the surface of HA low ST, a few round-shaped osteoblasts of both cell lines were observed, proving that this material did not promote cell adhesion and growth, which was in line with the MTT analysis done on the extracts.

## Discussion

Within this study, two types of the nanocomposite granules with different biopolymer content for bone regeneration were developed. Considering production method, it can be observed that composition of the chit/aga/nanoHA biomaterial was characterized by relatively larger total amount of biopolymers compared to the chit/curd/nanoHA. The chit/aga/nanoHA sample had also higher thermal stability of the biopolymer phase (chitosan/agarose). Therefore it was assumed that either agarose was more thermally stable than curdlan or chitosan/agarose matrix was characterized by a more crosslinked structure compared to the chitosan/curdlan one. In different studies, the thermal properties of chitosan with agarose^61,62^ and chitosan with curdlan^63,64^ blends were studied, but the results are equivocal. While Cao et al. found that a mix of chitosan and agarose, prepped at 95 °C, had lower thermal stability than pure chitosan, Ahmed found an opposite trend for materials prepped at 60 °C. The probable explanation for this phenomenon is the samples’ preparation temperature, which is known to affect the structure of polysaccharides^64–66^. In chitosan, self-crosslinking and the first steps of Maillard reactions could be initiated at temperatures exceeding 90 °C. This temperature corresponds to the one used by Cao et al.^61^ – it could be suggested that processing chitosan at temperatures exceeding 90 °C yields a highly self-crosslinked material, and blending it with other materials reduces its thermal stability and mechanical properties. In curdlan, heating above 80 °C yields the so-called high-set gel, wherein strong chemical bonds form a highly crosslinked sample. While Sun et al. found that mixing the chitosan and curdlan at 90 °C yielded a strong, uniform, and thermally stable gel, we found that this gel was still less stable than a chitosan/agarose blend^64^. It was likely that co-occurring mechanisms of chitosan and curdlan self-crosslinking reduced their ability to form interconnected blends, thus reducing their resistance to higher temperatures when compared to chitosan/agarose blends. This result was further supported by the microhardness analysis, wherein chit/curd/nanoHA was characterized by a lower Young’s modulus value than the chit/aga/nanoHA (Table 1) (5.2 vs. 8.2 GPa, respectively). It was also possible that the presence of 3,6-anhydro-L-galactose units in agarose created a material that was thermally sturdier than curdlan or chitosan and underwent carbonization with a higher yield of carbonaceous products. This is probable, as agarose has recently been gaining increasing attention as a possible source of green-derived carbon materials for different applications^53,67^.

Filling the bone defect, the biomaterial should imitate the mechanical strength of the surrounding tissue as much as possible. In addition to mechanical support, which is particularly important in the case of load-bearing tissues, the stiffness and microstructure of the surface may modulate cellular activity^68^. Young’s modulus is a parameter routinely used to determine the stiffness of a material^68^. The choice of the method used for the measurement, the location of the sampling site, and the condition of the patient, all may significantly affect Young’s modulus value of the human spongy bone^69^. In tension, compression, or bending tests, it was estimated that Young’s modulus of healthy human spongy bone was in the range of 2.2 ± 0.5 GPa – 16.2 ± 2.5 GPa. However, using the ultrasonic and nanoindentation methods, this value was in the range of 10 ± 1.3 GPa – 17.5 ± 1.1 GPa and 1.3 ± 0.2–22.3 ± 3 GPa, respectively^69^. It is worth noting that the values obtained in tension, compression, or bending tests are more accurate, but the sample size and shape requirements limit their applicability. In this study, a microhardness test was employed to assess Young’s modulus of the produced nanocomposite granules. The test revealed that chit/aga/nanoHA sample had slightly higher Young’s modulus (but without statistical significance) compared to the HA low ST and the chit/curd/nanoHA granules (Table 1). This difference may have resulted from the high flexibility of the curdlan component after thermal gelation. It is known that curdlan forms a firm and flexible gel after heating^70^. These results are also in line with the TGA analysis (Fig. 2c), wherein chit/aga/nanoHA was characterized by a higher thermal stability, suggesting a more crosslinked structure. On the other hand, commercial HA high ST granulate was the most rigid material with Young’s modulus value of 82.0 GPa. The high-temperature sintering technique is a production method that results in a material with large grains, low porosity, and high crystallinity. These parameters drastically reduce the flexibility of the material^9^. Zima et al., in turn, indicated an improvement in the compressive strength of hybrid hydroxyapatite-chitosan granules compared to hydroxyapatite granules. Granules with 17% and 23% chitosan content were characterized by a 12- and 16-fold increase in compressive strength, respectively, compared to granules containing only hydroxyapatite^71^. Both too-low and too-high mechanical strengths are not beneficial for biomaterials dedicated to bone treatment. Superior Young’s modulus of dense ceramics compared to natural bone can lead to stress shielding, resulting in bone resorption due to insufficient loading^72^. In turn, biomaterial with an inferior Young’s modulus may lead to material failure or an excessive bone formation and packing, due to too high loading^73,74^.

In regenerative medicine of bone tissue, biomaterials with a porous microstructure are preferred. The pores present in the biomaterial provide space for cell migration and proliferation, stimulate their differentiation, and ensure free transport of nutrients, waste products of metabolism, and gases within the biomaterial^75^. However, it should be taken into account that the increase in porosity negatively affects the mechanical parameters and complicates the implant production process^75^. In the available literature, pores are divided according to their diameter into three groups: micropores, mesopores, and macropores. However, scientists assigned different ranges of values to each of the categories. Kazimierczak et al. divided the pores as follows: micropores < 2 μm, mesopores 2–50 μm, and macropores > 50 μm^33^. Morejon et al. classified nanopores below 0.1 μm, micropores in the range of 0.1–10 μm and macropores above 100 μm^76^, while according to Abbasi et al., micropores are below 10 μm and macropores above 50 μm^77^. Developed nanocomposite granules showed relatively high SSA (near 30 m²/g) and high porosity (near 50%) that was within the range of cancellous bone porosity (30–90%)^54^. Both types of the produced granules exhibited a large share of micropores in their microstructure. However, chit/aga/nanoHA biomaterial had dominant content of macropores, whereas chit/curd/nanoHA granules revealed the dominant presence of mesopores. Kołodziejska et al. produced, among others, composite granules based on hydroxyapatite enriched with Mg^2+^, CO_3_^2−^ and Zn^2+^ ions, type I collagen, alginate, chitosan, and sericin. Kołodziejska et al. also noticed that mesopores dominated the microstructure of their biomaterials^78^. It should be noted that the porosity and SSA of implantable biomaterials directly affect their biocompatibility and bioresorbability. The presence of macropores promotes the adhesion and proliferation of osteoblasts and the formation of new blood vessels within the implant. On the other hand, microporosity, closely related to the increase in SSA, enlarges the ion exchange with a microenvironment and thus increases the biomaterial’s bioactivity. Moreover, the presence of micropores enhances the access of biomaterial to body fluids, facilitating its biodegradation^9^. Interestingly, it was proven that mesopores, similarly to micropores, contribute to the increase in SSA of the biomaterial, participating in ion exchange with the microenvironment, thus improving its osteoconductivity and bioactivity^79^. In addition, mesopores could serve as reservoirs of bioactive factors and nutrients, constituting effective carriers of, e.g. growth factors, ensuring their release over a long period of time^80^. According to the available literature, the use of high temperatures during the sintering process of HA causes a large grain growth, as a result of the strong binding of the powder particles to each other, reducing the volume of voids, leaving only residual porosity^4,81^. This quality is then inherited by HA high ST–based biomaterials^7,8,82^. On the other hand, the application of low sintering temperatures results in the creation of ceramics with significantly higher porosity and SSA^7,8^. In the current research, the use of a polymer matrix as a binder for high ST nanoHA, along with a specific production method (application of porogen and the lyophilization process), resulted in a highly porous microstructure (comparable to HA low ST), and a relatively high SSA (approx. 12-fold higher compared to HA high ST).

The surface topography of the material is an important factor that influences the effectiveness of osseointegration^83^. The rough surface increases the area of contact between the material and the bone, which is associated with better adhesion of both surfaces. There are studies suggesting that osteogenesis occurs faster on rough surfaces^84^. In addition, the irregular surface topography, rich in elevations or cracks, supports the adsorption of proteins mediating cell adhesion and facilitates cellular adhesion (including osteoblasts)^85^. Moreover, the roughness with a certain morphology may have a positive effect on the bioactivity of the biomaterial^86^. Both produced nanocomposites (chit/aga/nanoHA and chit/curd/nanoHA) had very rough surface comparable to HA high ST (Fig. 4b). Consequently, produced biomaterials showed high bioactivity and biocompatibility. The bioactivity of the biomaterial is an important property that determines the formation of a bond between the implantable biomaterial and the host bone tissue^87^. In contact with body fluids, bioactive material induces the formation of an apatite coating on its surface. In vitro, bioactivity can be evaluated by incubating the biomaterial in SBF and evaluating the as-formed deposits^88,89^. SEM analysis revealed that incubation of nanocomposite granules in SBF resulted in the formation of apatite-like crystals with spherical, hemispherical, or cauliflower-like morphology (Fig. 4c). Apatite with a similar spherical morphology was also noted on the surface of silicon-based bioceramics and polymer/hydroxyapatite composite^33,90^. Also, Zima et al. described the formation of cauliflower-like microspheres of apatite on the surface of hydroxyapatite/chitosan granules^71^. The performed analysis indicated that the presence of biopolymers did not reduce the bioactivity of the HA-based nanocomposites. On the contrary, by increasing the porosity and SSA, they stimulated the formation of apatite with Ca/P ratio closer to stoichiometric, when compared to the one forming on HA high ST (1.8 vs. 2.1). This suggests that the presence of biopolymers on the surface of HA sintered at high temperatures speed up the formation of crystalline apatite.

In order to create space for the newly formed bone, biomaterial should undergo gradual biodegradation (degradation mediated by biological agents such as enzymes, and cells), bioerosion (physical and chemical processes under physiological conditions), and bioresorption (removal of biomaterial by cellular activity)^91^. The rate of implant disintegration should be adjusted to the rate of regeneration of the damaged tissue^92^. Cells may be involved in the bioresorption/biodegradation of calcium phosphate-based biomaterials by phagocytosing small particles (< 10 μm), altering the pH of the microenvironment, or releasing enzymes to lyse large particles (> 100 μm). Depending on the particle size and chemical composition, cells responsible for biomaterial bioresorbability are: monocytes, macrophages, or multinucleated giant cells. Bioresorption with the participation of osteoclasts proceeds mainly by lowering the pH^93^. The biomaterial parameters affecting the rate of biodegradation include composition, porosity, crystallinity, and SSA^94^. The biodegradation of granules was assessed in three different environments: collagenase I and lysozyme solution, simulating the microenvironment of enzyme-mediated bone bioresorption; HCl solution at pH 4.5, simulating an acidified microenvironment during osteoclast-mediated bone resorption, and 0.9 w/v % NaCl solution at pH 5.9 that served as a non-aggressive isotonic microenvironment for evaluation of material stability. Importantly, both nanocomposite granules showed high bioresorbability and degraded significantly faster compared to HA high ST and HA low ST (Fig. 5a). Herein, surprising is a slightly higher mass loss (10%) recorded for the HA high ST incubated in NaCl compared to HA low ST. Based on the available literature, sodium ions might participate in HA degradation, and one possible mechanism is ionic substitution^95,96^. However, at this stage, we were not able to explain why HA high ST degraded faster than the HA low ST. Importantly, depending on the ions present in the surrounding fluid, the solubility of HA might either be speed up or slowed down. Hence, it was assumed that HA low ST either repulses Na^+^ ions and attracts the Cl¯ ions, mitigating the ionic substitution or there are enough negatively charged species on the surface of HA low ST for the Na^+^ ions to attach without the need of substituting Ca^2+^ ions. Both mechanisms, that might happen simultaneously at different regions of the sample, can be responsible for the reduction of the solubility of HA low ST in the NaCl solution. Interestingly, incubation of the chit/curd/nanoHA in NaCl and enzymes resulted in 20% and 30% mass drop, respectively, which was not consistent with the values found in TGA analysis (16%), suggesting degradation of both phases: polymeric and ceramic (nanoHA). In TGA and microstructural evaluations, chit/curd blend was suggested to form a less crosslinked and more porous matrix, it also had a higher share of chitosan in its structure. As such, it was more susceptible to biodegradation induced by the enzymes and by the chloride ions. Evaluating the chemical structure of chitosan, agarose, and curdlan, and analyzing our previous results^32,47^, one can tell that the structure of the chit/curd mix was much more prone to biodegradation, especially in the presence of lysozyme, which was able to break most of the bonds in its backbone. Apparently, the products of this process participated in the degradation of HA, possibly by lowering the pH from the broken peptide bonds (affected by collagenase and by the presence of Na^+^ and Cl^−^ ions). Interestingly, both polymer blends were similarly affected by the HCl, with comparable weight losses of 25% - in these conditions, both blends degraded by similar routes, and their degradation products were similar, affecting the HA to the same extent. Within this study it was also proven that HA nanoparticles released from the developed nanocomposite granules during degradation process may be easily bioresorbed by macrophages, mouse preosteoblasts, and human fetal osteoblasts (Fig. 5b-d). In most scientific works, the bioresorption process of the biomaterial was studied in vivo. C. M. Müller-Mai et al. placed HA implants in the trabecular bone of the distal femur of female chinchilla rabbits for 84 days. The authors also observed phagocytized HA implant particles inside macrophages on the surface of the biomaterial^97^.

The ability of a biomaterial to absorb liquid is an important factor in determining the availability of nutrients for the cells inhabiting the material. This provides an appropriate environment for cells to proliferate and differentiate and affects their distribution^98^. Before the biomaterial implantation, soaking in patients’ blood or plasma is often required. Therefore, the short time required to reach biomaterial equilibrium is important to minimize the risk of its contamination and postoperative complications due to bacterial infection^33^. Performed experiments with use of blood plasma and PBS showed that both chit/aga/nanoHA and chit/curd/nanoHA granules absorbed a large volume of liquids, reaching the equilibrium within approx. 30 s (Fig. 6a and b). This is due to the fact that both of the blends used for the granules production are hydrogels in nature – their high absorption capacity has already been proven in our previous studies^32,47^. In line with the above-mentioned results that suggested the chit/curd matrix to be less crosslinked (TGA and mechanical properties), this material was also found to absorb significantly more liquids than chit/aga. Curdlan is also known to have a very high water-holding capacity and thus is commonly used as a food additive^99^. Hence, chit/curd/nanoHA not only absorbs more fluids but also reaches the equilibrium faster, probably due to higher shares of water bonding functional groups available on its surface, which is further enhanced by higher porosity reported in this material. Similarly, Kołodziejska et al. showed high water absorption capacity of the composite HA-based granules, especially in the case of those containing chitosan. Nevertheless, delayed swelling was observed, which may be unfavorable in the case of bone implants^78^. Before the implantation procedure, biomaterials are often soaked in the patient’s blood plasma to reach their target size. Prolonged fluid absorption time may expose the biomaterial to contamination with microorganisms^38^.

Biocompatibility understood as the lack of a negative impact on the proper functioning of host cells, is an essential feature of the biomaterial^9^. The cytotoxicity of the granules was assessed indirectly according to ISO 10993-5 (2009) standard for medical devices by colorimetric MTT test and directly by culturing the cells on the surface of the granules with further Live/Dead staining assay. In vitro studies clearly proved non-toxicity of the nanocomposite granules and HA high ST towards mouse preosteoblasts (MC3T3-E1) and human fetal osteoblasts (hFOB 1.19) (Fig. 7a-d). However, a lower number of hFOB 1.19 cells and of a less spread-out morphology were observed on the surface of chit/curd/nanoHA. It can thus be suggested that this material, while non-cytotoxic, was less supportive for the osteoblasts’ adhesion and proliferation after 3 days of culture. The possible explanation was a too-high hydrophilicity (as proven by quickly reaching absorption equilibrium (Fig. 6), which can mitigate the adsorption of proteins that are important for consecutive cellular adhesion^100,101^. Moreover, despite the high viability of hFOB 1.19 cells grown on the chit/curd/nanoHA, their morphology was negatively affected, indicating their worse adhesion. Klimek et al. also investigated the cytotoxicity of a scaffold with similar composition, i.e. made of β-1,3-glucan (curdlan) and hydroxyapatite, in direct contact with MC3T3-E1 and hFOB 1.19 cells. Similarly, CLSM microscopy analysis performed after Live/Dead staining showed that tested curdlan/HA biomaterial was more supportive to the adhesion and growth of MC3T3-E1 cells compared to the hFOB 1.19 osteoblasts^102^. Perhaps the process of the human fetal osteoblasts adhesion is more challenging than the attachment of mouse preosteoblasts. Kolmas et al. also assessed, among others, hydroxsyapatite-alginate composite granules containing selenium and sodium risedronate in terms of cytotoxicity. The studies showed that the produced biomaterial significantly reduced the viability of both osteosarcoma cancer cells and normal fetal osteoblasts^103^. Kołodziejska et al. composite granulates, in turn, were non-toxic to the MG-63 cell line (osteosarcoma). However, it should be noted that the authors used in their research an extract prepared by incubation of 50 mg of the biomaterial in 1 mL of the medium, whereas in this study the extract prepared at the ratio of 100 mg sample per 1 mL of the medium was used^78^. Unsurprisingly, HA low ST revealed cytotoxicity in vitro (Fig. 7a-d), which is in agreement with the literature data of other authors who indicated the toxic nature of biomaterials based on hydroxyapatite sintered at low temperatures^6,11,12,14,104^. HA sintered at low temperatures was found to reduce the levels of calcium and phosphate ions in the culture medium – these ions are important for cells’ survival^9,104^. Ions reduction was associated with high SSA of these materials, which significantly affected the rate of ion exchange (ion reactivity) with the medium. The ions taken from the medium were probably incorporated into the structure of the newly formed apatite layer on the surface of biomaterial^9,105^. The presence of calcium and phosphate ions in the microenvironment is essential for the proper functioning of adherent cells, especially osteoblasts. Therefore, too high ionic reactivity of the biomaterial may adversely affect its biocompatibility^9,104^. Based on the obtained results, it can be concluded that both tested granulates were non-toxic to mouse preosteoblasts and human fetal osteoblasts. However, higher biocompatibility was revealed in the case of chit/aga/nanoHA granules, which supported cell adhesion and growth.

## Conclusion

In this study, it was demonstrated that HA-based biomaterial characterized by both biocompatibility and highly porous microstructure may be produced using mixtures of polysaccharides, a porogenic agent, and a freeze-drying process. Two variants of granules were created from the combination of hydroxyapatite nanopowder sintered at high temperature, and a polymer matrix made of chitosan/agarose, and chitosan/curdlan. Both types of nanocomposites showed high biocompatibility that was even better than that observed for the commercial HA granules sintered at high temperatures. Furthermore, the obtained nanocomposite granules showed improved microstructural properties similar to the granules sintered at low temperatures. It was concluded that due to higher biocompatibility and more optimal mechanical parameters, granules made of chitosan, agarose, and nanoHA show greater biomedical potential than those containing chitosan/curdlan matrix.

## Electronic supplementary material

Below is the link to the electronic supplementary material.

Supplementary Material 1

## Data Availability

The datasets generated during and/or analysed during the current study are available in the Mendeley Data repository, DOI: 10.17632/bcy87d4dsb.1.

## Abbreviations

ARS: Alizarin Red S
ATCC: American Type Culture Collection
β-TCP: β-tricalcium phosphate
chit/aga/nanoHA: Chitosan/agarose/nanoHA
chit/curd/nanoHA: Chitosan/curdlan/nanoHA
CLSM: Confocal laser scanning microscope
DMEM: Dulbecco’s Modified Eagle’s Medium
EDS: Energy dispersive spectroscopy
FBS: Fetal bovine serum
FTIR: Fourier transform infrared spectroscopy
HA high ST: Hydroxyapatite granules sintered at high temperature of 1250 °C
HA low ST: Hydroxyapatite granules sintered at low temperature of 400 °C
HA: Hydroxyapatite
KBr: Potassium bromide
nanoHA: Nanohydroxyapatite
PBS: Phosphate-buffered saline
PBS+: Phosphate-buffered saline with calcium and magnesium
PI: Propidium iodide
SBF: Simulated body fluid
SD: Standard deviation
SEM: Scanning electron microscope
SSA: Specific surface area
TGA: Thermogravimetric analysis
XRD: X-ray diffraction
