# Supplementary material for: Biocompatible nanocomposite hydroxyapatite-based granules with increased specific surface area and bioresorbability for bone regenerative medicine applications
Source: Sci Rep. 2024 Nov 15;14:28137. doi: 10.1038/s41598-024-79822-0 (PMC11568164; doi:10.1038/s41598-024-79822-0)
Supplement: Supplementary file 1 — Supplementary Material 1 [file 41598_2024_79822_MOESM1_ESM.pdf]

Biocompatible nanocomposite hydroxyapatite-based granules with increased specific surface area and bioresorbability for bone regenerative medicine applications

Marta Trzaskowska[1], Vladyslav Vivcharenko[1], Aleksandra Benko[2], Wojciech Franus[3], Tomasz Goryczka[4], Adrian Barylski[4], Krzysztof Palka[5], Agata Przekora[1]*

[1] Department of Tissue Engineering and Regenerative Medicine, Medical University of Lublin, Chodzki 1, 20-093 Lublin, Poland

[2] AGH University of Science and Technology, Faculty of Materials Science and Ceramics, Mickiewicza 30, 30-059 Krakow, Poland

[3] Department of Construction Materials Engineering and Geoengineering, Lublin University of Technology, Nadbystrzycka 38 D, 20-618 Lublin, Poland

[4] Institute of Materials Engineering, University of Silesia in Katowice, 75 Pułku Piechoty 1A, 41-500 Chorzów, Poland

[5] Department of Materials Engineering, Lublin University of Technology, Nadbystrzycka 36, 20-618 Lublin, Poland

* corresponding author: agata.przekora@umlub.pl

Supplementary Data 1

XRD diffractograms obtained for HA low ST and commercial hydroxyapatites (HA high ST and nanoHA)

HA low ST (produced by wet chemical precipitation method)

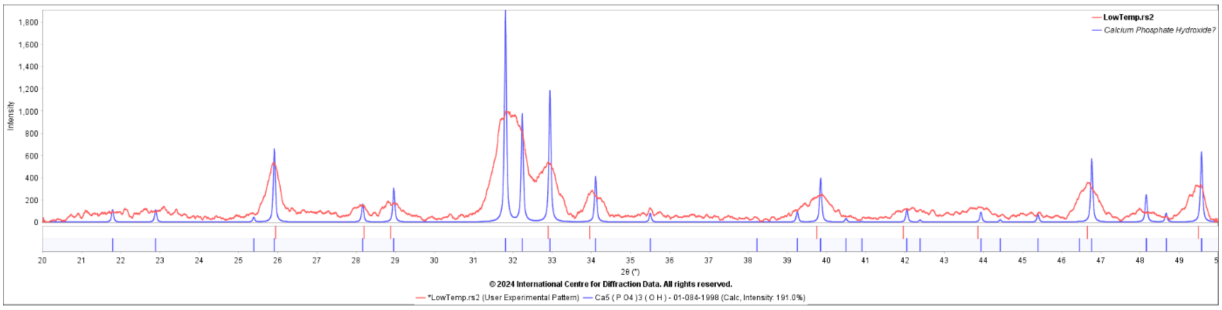

HA high ST (HA BIOCER, Chema Elektromet Rzeszow)

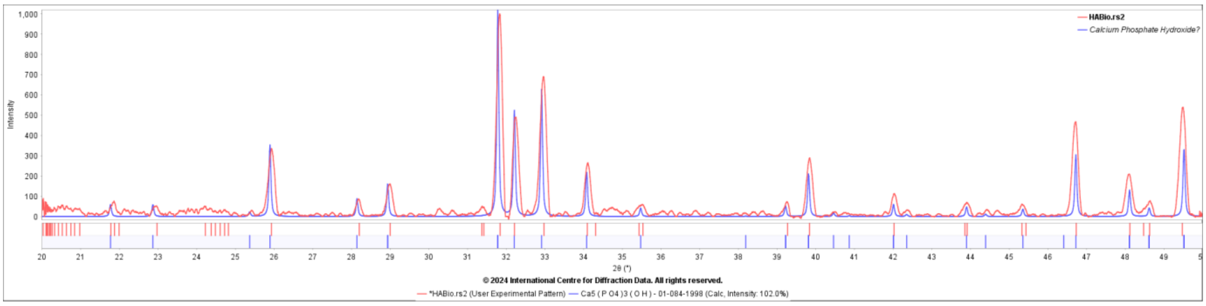

nanoHA (Sigma-Aldrich Chemicals)

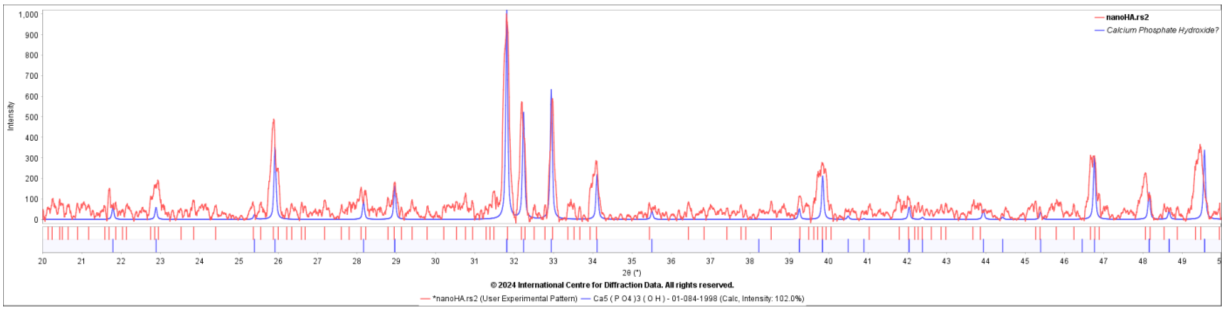

Supplementary Data 2

Maximized and superimposed FTIR spectra

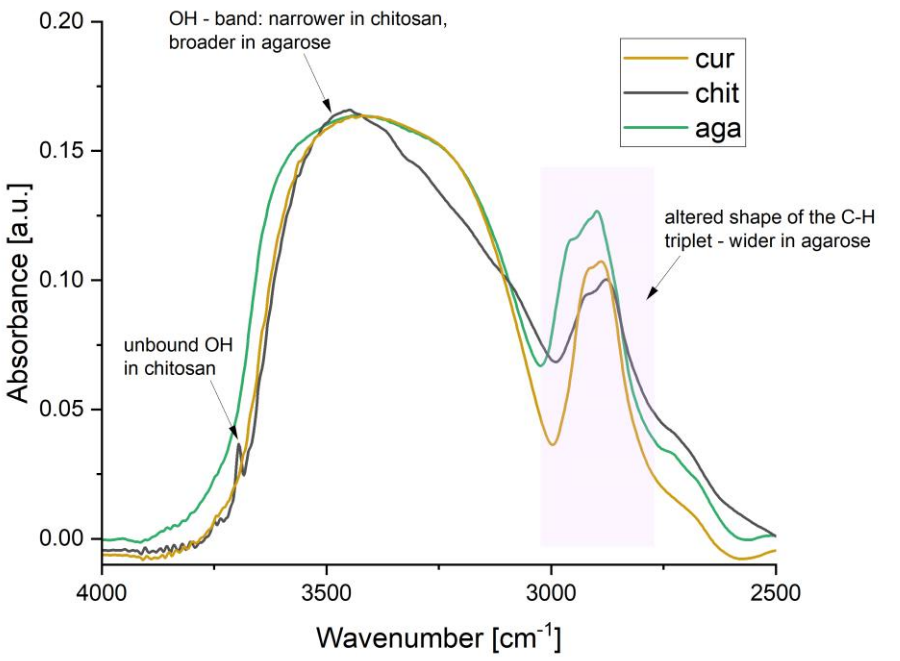

Highlighted differences in the spectra of 3 polysaccharides used in this study, presented in the 4000 - 2500 cm^{-1} region.

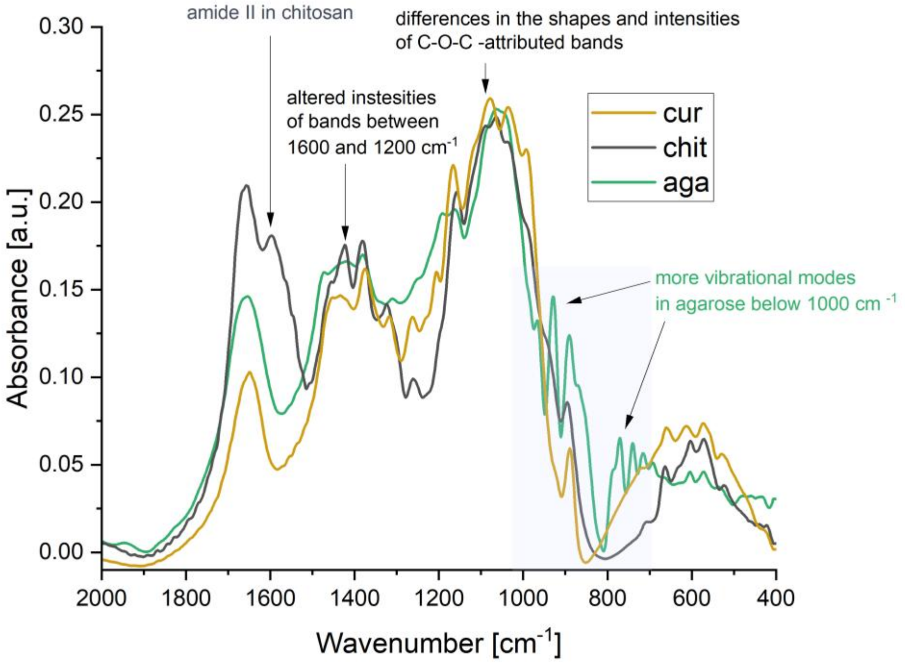

Highlighted differences in the spectra of 3 polysaccharides used in this study, presented in the 4000 - 2500 cm^{-1} region.

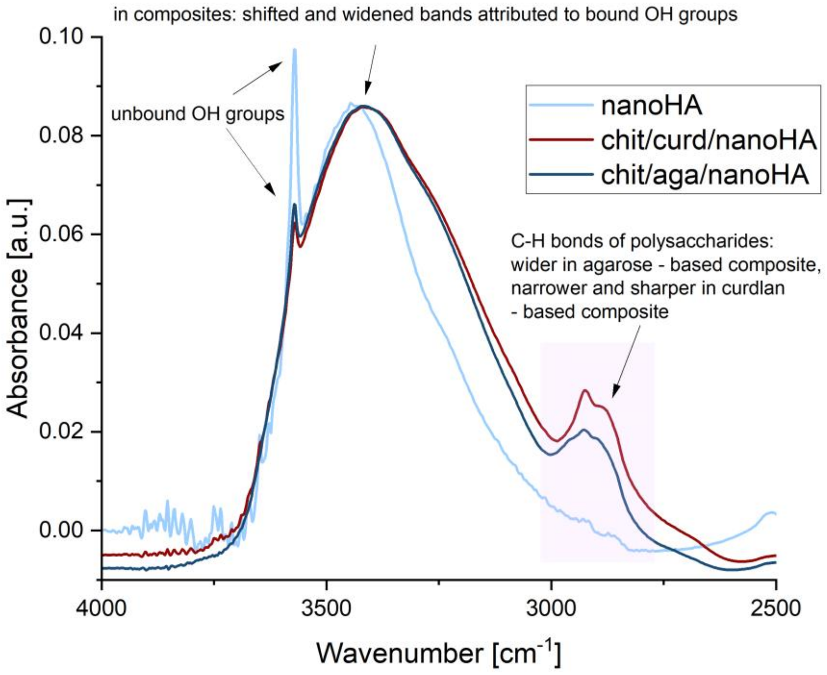

Highlighted differences in the spectra of 2 composites and nanoHA, presented in the 4000 - 2500 cm^{-1} region.

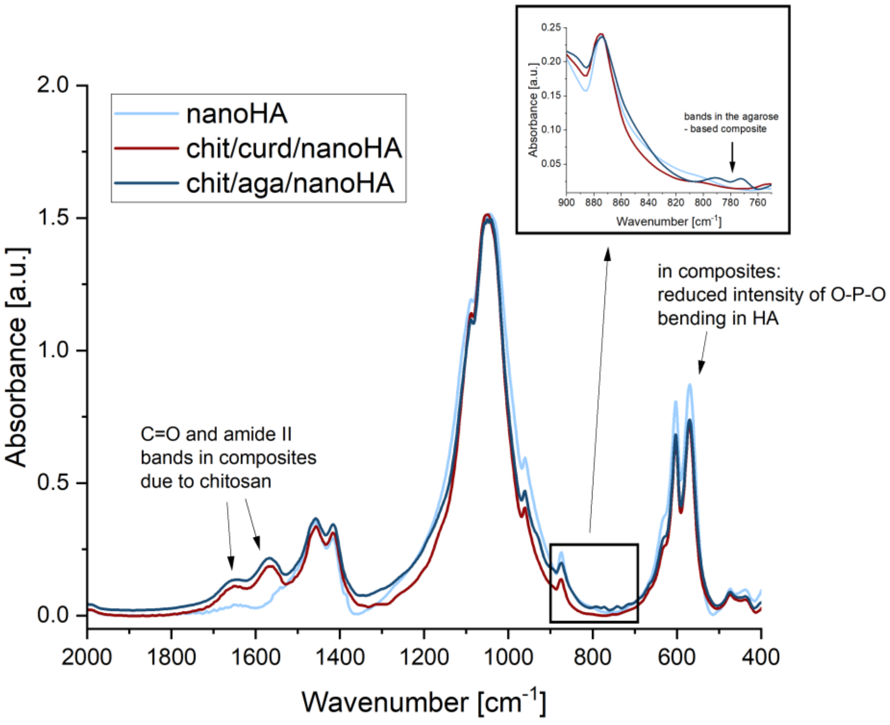

Highlighted differences in the spectra of 2 composites and nanoHA, presented in the 2000 - 400 cm^{-1} region. The inset in the picture presents a maximize 900 – 750 cm^{-1} region, which reveals some bands characteristic of agarose.

Supplementary Data 3

Ca/P atomic ratio before and after incubation in SBF

Sample	Before immersion in SBF<br>Mean ± SD	After immersion in SBF<br>Mean ± SD
chit/aga/nanoHA	2.61 ± 0.26	1.82 ± 0.16
chit/curd/nanoHA	2.78 ± 0.30	1.81 ± 0.06
HA low ST	1.67 ± 0.03	1.74 ± 0.03
HA high ST	1.92 ± 0.09	2.11 ± 0.27

Exemplary EDS data before incubation in SBF

chit/aga/nanoHA sample (spot 1)

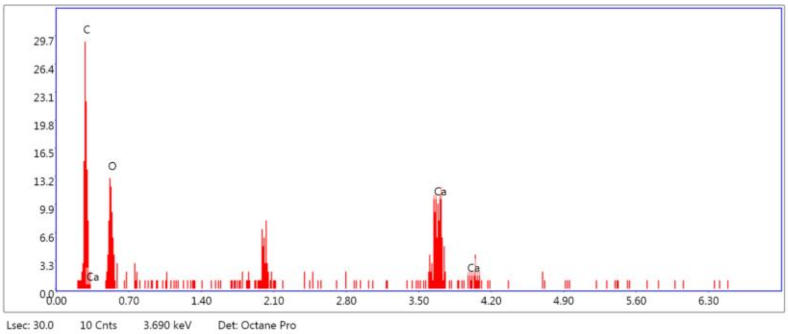

Element	Weight %	Atomic %	Net Int.	Error %	Kratio	Z	A	F
C K	11.68	26.26	3.32	18.65	0.1084	1.2201	0.7607	1.0000
O K	14.06	23.72	2.17	25.41	0.0535	1.1493	0.3312	1.0000
CaK	74.26	50.02	3.95	17.45	0.6932	0.9314	1.0014	1.0008

chit/aga/nanoHA sample (spot 2)

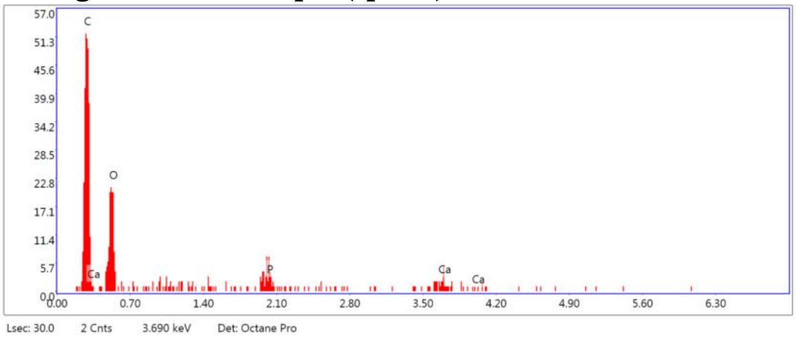

Element	Weight %	Atomic %	Net Int.	Error %	Kratio	Z	A	F
C K	45.84	63.06	9.19	13.97	0.3442	1.0955	0.6855	1.0000
O K	22.02	22.74	3.62	24.41	0.1024	1.0290	0.4518	1.0000
P K	7.88	4.20	1.34	36.04	0.0679	0.8624	0.9864	1.0131
CaK	24.26	10.00	1.00	42.97	0.2010	0.8221	1.0021	1.0056

chit/curd/nanoHA sample (spot 1)

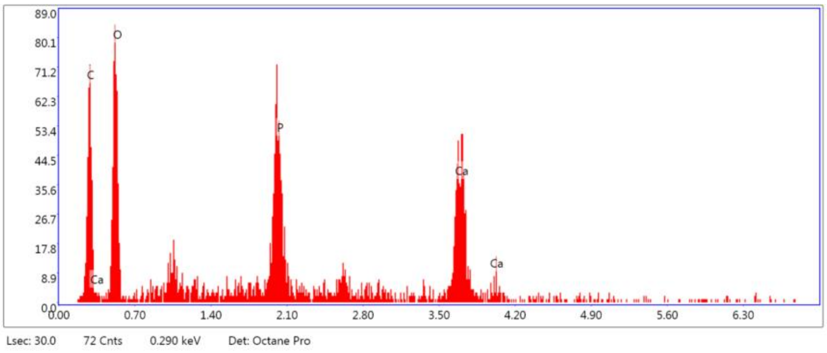

Element	Weight %	Atomic %	Net Int.	Error %	Kratio	Z	A	F
C K	8.10	18.35	8.88	15.84	0.0523	1.2228	0.5278	1.0000
O K	15.80	26.89	14.97	15.49	0.0665	1.1517	0.3655	1.0000
P K	15.45	13.57	18.54	12.40	0.1483	0.9709	0.9733	1.0159
CaK	60.65	41.19	17.84	14.25	0.5643	0.9320	0.9968	1.0014

chit/curd/nanoHA sample (spot 2)

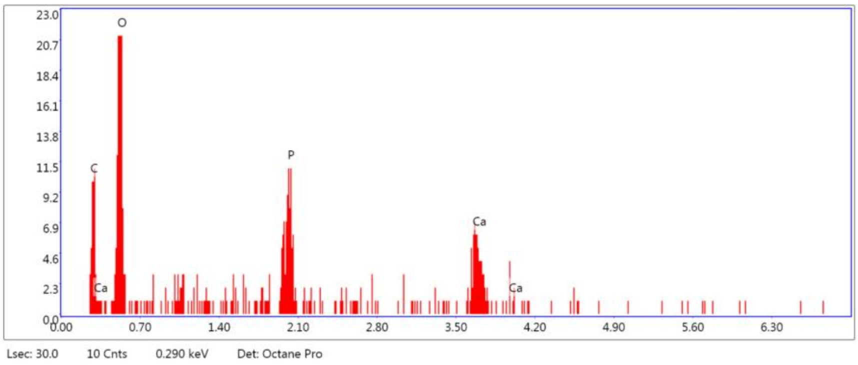

Element	Weight %	Atomic %	Net Int.	Error %	Kratio	Z	A	F
C K	5.84	12.12	0.93	37.97	0.0365	1.2002	0.5207	1.0000
O K	28.21	43.97	4.49	18.95	0.1322	1.1298	0.4149	1.0000
P K	15.71	12.65	2.78	24.94	0.1475	0.9511	0.9737	1.0139
CaK	50.24	31.26	2.18	31.28	0.4575	0.9114	0.9970	1.0021

HA low ST – reference sample

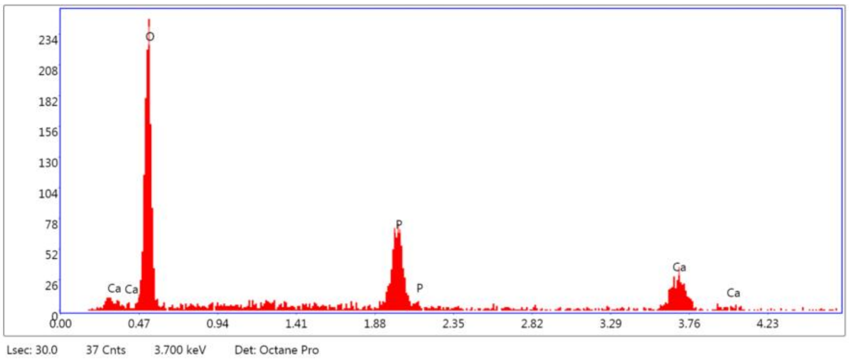

Element	Weight %	Atomic %	Net Int.	Error %	Kratio	Z	A	F
O K	38.07	58.47	49.91	11.01	0.2007	1.1239	0.4692	1.0000
P K	19.73	15.66	25.37	9.68	0.1838	0.9455	0.9741	1.0111
CaK	42.20	25.87	13.32	14.43	0.3814	0.9052	0.9957	1.0028

HA high ST – reference sample

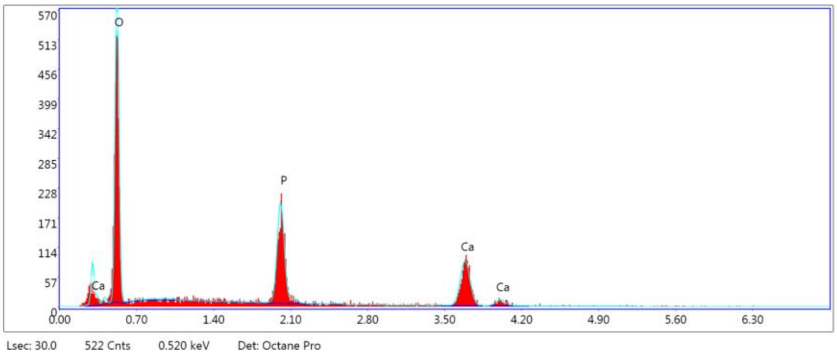

Element	Weight %	Atomic %	Net Int.	Error %	Kratio	Z	A	F
O K	34.59	54.96	94.47	10.38	0.1733	1.1322	0.4426	1.0000
P K	18.98	15.58	53.99	8.65	0.1781	0.9530	0.9730	1.0120
CaK	46.43	29.45	32.44	10.72	0.4231	0.9130	0.9958	1.0024

Exemplary EDS data after incubation in SBF

chit/aga/nanoHA sample (spot 1)

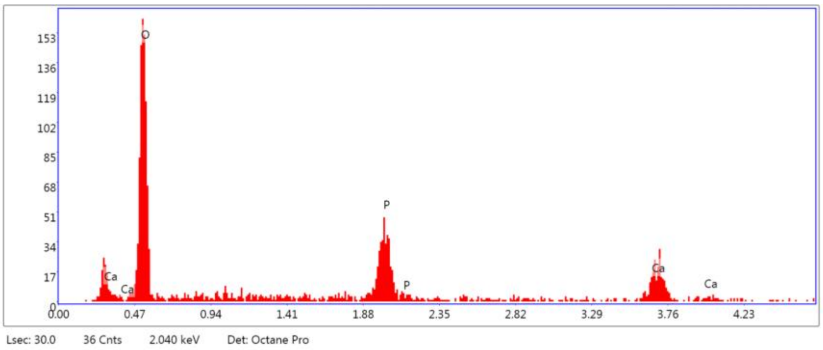

Element	Weight %	Atomic %	Net Int.	Error %	Kratio	Z	A	F
O K	37.11	57.61	26.14	12.10	0.1944	1.1264	0.4650	1.0000
P K	18.79	15.06	13.08	13.17	0.1754	0.9478	0.9740	1.0117
CaK	44.10	27.33	7.54	20.69	0.3997	0.9076	0.9960	1.0026

chit/aga/nanoHA sample (spot 2)

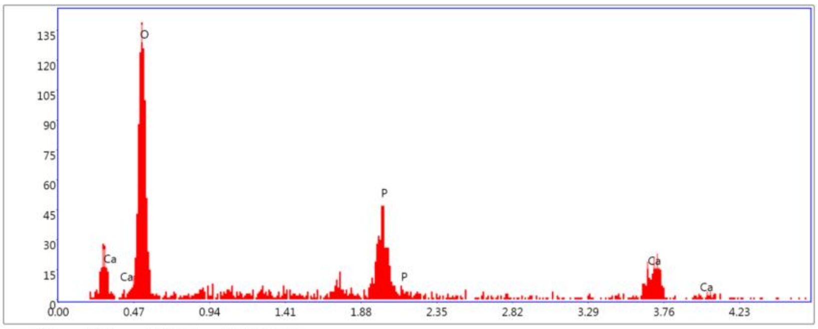

Element	Weight %	Atomic %	Net Int.	Error %	Kratio	Z	A	F
O K	39.99	60.43	29.76	11.73	0.2162	1.1198	0.4828	1.0000
P K	19.00	14.83	13.45	12.78	0.1763	0.9418	0.9745	1.0111
CaK	41.02	24.74	7.13	18.97	0.3693	0.9015	0.9959	1.0029

chit/curd/nanoHA sample (spot 1)

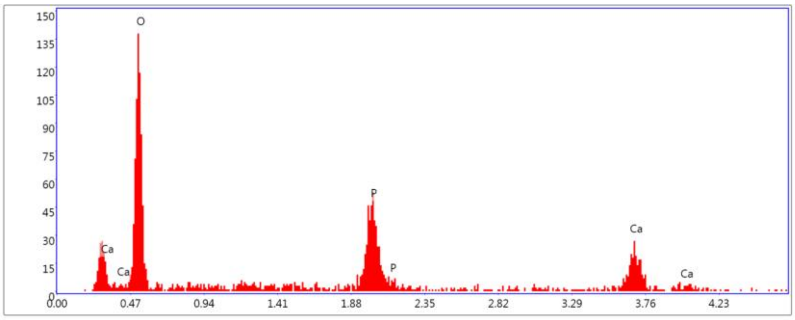

Element	Weight %	Atomic %	Net Int.	Error %	Kratio	Z	A	F
O K	33.98	54.03	26.81	12.30	0.1757	1.1328	0.4565	1.0000
P K	21.77	17.88	17.31	10.03	0.2045	0.9534	0.9747	1.0109
CaK	44.25	28.09	8.63	15.74	0.4033	0.9133	0.9952	1.0025

chit/curd/nanoHA sample (spot 2)

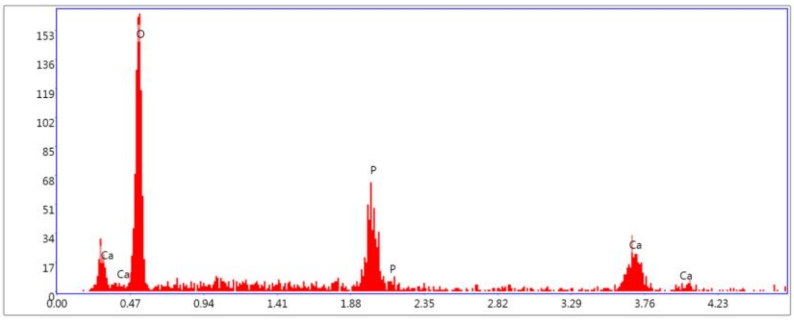

Element	Weight %	Atomic %	Net Int.	Error %	Kratio	Z	A	F
O K	36.23	56.70	34.70	11.73	0.1878	1.1284	0.4595	1.0000
P K	18.86	15.25	18.08	11.50	0.1765	0.9496	0.9739	1.0118
CaK	44.91	28.05	10.56	16.79	0.4078	0.9095	0.9961	1.0025

HA low ST – reference sample

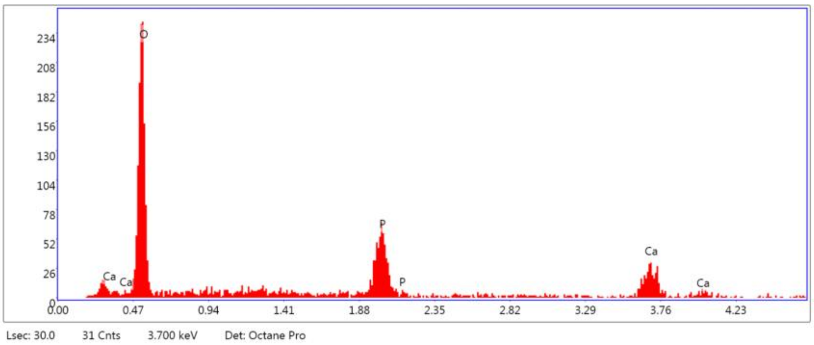

Element	Weight %	Atomic %	Net Int.	Error %	Kratio	Z	A	F
O K	40.52	60.98	46.49	10.94	0.2175	1.1187	0.4799	1.0000
P K	18.65	14.50	20.51	10.46	0.1728	0.9408	0.9739	1.0111
CaK	40.82	24.52	11.02	14.99	0.3672	0.9005	0.9961	1.0029

HA high ST – reference sample

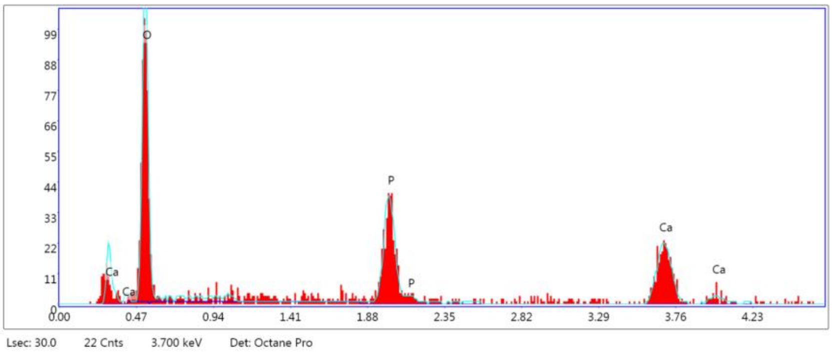

Element	Weight %	Atomic %	Net Int.	Error %	Kratio	Z	A	F
O K	31.04	51.22	20.70	13.71	0.1500	1.1411	0.4235	1.0000
P K	17.34	14.78	12.59	13.38	0.1643	0.9610	0.9725	1.0134
CaK	51.62	34.01	9.20	17.66	0.4748	0.9214	0.9960	1.0020
